# DAMP Molecule S100A9 Acts as a Molecular Pattern to Enhance Inflammation during Influenza A Virus Infection: Role of DDX21-TRIF-TLR4-MyD88 Pathway

**DOI:** 10.1371/journal.ppat.1003848

**Published:** 2014-01-02

**Authors:** Su-Yu Tsai, Jesus A. Segovia, Te-Hung Chang, Ian R. Morris, Michael T. Berton, Philippe A. Tessier, Mélanie R. Tardif, Annabelle Cesaro, Santanu Bose

**Affiliations:** 1 Department of Microbiology and Immunology, The University of Texas Health Science Center at San Antonio, San Antonio, Texas, United States of America; 2 Axe Maladies Infectieuses et Immunitaires, Centre de Recherche du CHU de Québec, and Faculté de Médecine, Université Laval, Quebec, Canada; Harvard Medical School, United States of America

## Abstract

Pathogen-associated molecular patterns (PAMPs) trigger host immune response by activating pattern recognition receptors like toll-like receptors (TLRs). However, the mechanism whereby several pathogens, including viruses, activate TLRs via a non-PAMP mechanism is unclear. Endogenous “inflammatory mediators” called damage-associated molecular patterns (DAMPs) have been implicated in regulating immune response and inflammation. However, the role of DAMPs in inflammation/immunity during virus infection has not been studied. We have identified a DAMP molecule, S100A9 (also known as Calgranulin B or MRP-14), as an endogenous non-PAMP activator of TLR signaling during influenza A virus (IAV) infection. S100A9 was released from undamaged IAV-infected cells and extracellular S100A9 acted as a critical host-derived molecular pattern to regulate inflammatory response outcome and disease during infection by exaggerating pro-inflammatory response, cell-death and virus pathogenesis. Genetic studies showed that the DDX21-TRIF signaling pathway is required for S100A9 gene expression/production during infection. Furthermore, the inflammatory activity of extracellular S100A9 was mediated by activation of the TLR4-MyD88 pathway. Our studies have thus, underscored the role of a DAMP molecule (i.e. extracellular S100A9) in regulating virus-associated inflammation and uncovered a previously unknown function of the DDX21-TRIF-S100A9-TLR4-MyD88 signaling network in regulating inflammation during infection.

## Author Summary

The lung disease severity following influenza A virus (IAV) infection is dependent on the extent of inflammation in the respiratory tract. Severe inflammation in the lung manifests in development of pneumonia. Therefore, it is very critical to identify cellular factors and dissect the molecular/cellular mechanism controlling inflammation in the respiratory tract during IAV infection. Knowledge derived from these studies will be instrumental in development of therapeutics to combat the lung disease associated with IAV infection. Towards that end, in the current study we have identified a cellular factor S100A9 which is responsible for enhanced inflammation during IAV infection. In addition, we have characterized a signal transduction pathway involving various cellular receptors and signaling adaptors that are involved in mediating S100A9-dependent inflammatory response. Thus, our studies have illuminated a cellular/molecular mechanism that can be intervened by therapeutics to reduce and control IAV-associated lung inflammatory disease like pneumonia.

## Introduction

Pathogen-associated molecular patterns (PAMPs) are molecular signatures of pathogens which facilitate induction of the host immune response [1], [2]. PAMPs activate cellular pattern-recognition-receptors (PRRs) such as toll-like receptors (TLRs) to induce immunity [1], [2]. Wide arrays of pathogens activate PRRs in the absence of PRR-specific PAMPs. It is thought that during infection cellular factors can activate PRRs and thus indirectly fulfill the function of PAMPs. The mechanism regulating the activity and function of non-PAMP dependent immune response during virus infection is still an enigma. Damage-associated molecular patterns (DAMPs), which are molecules produced from damaged or dead cells induce an inflammatory response in paracrine fashion via TLR activation [3]. However, whether DAMPs can function as a host-derived molecular pattern during virus infection is not known. In this study, we determined that during influenza A (IAV) virus infection, S100A9 protein (also known as Calgranulin B or MRP-14), which is classified as a DAMP, is released from undamaged infected cells to activate the TLR4/MyD88 pathway for induction of innate and inflammatory responses against IAV. Thus, we have identified extracellular S100A9 as a critical host-derived molecular pattern during IAV infection. This protein has an essential role in enhancing the inflammatory response, which culminates in exacerbated IAV pathogenesis and lung disease.

Influenza A virus (IAV) is a negative-sense, single-stranded RNA virus that causes severe respiratory tract infection [4]–[6]. Infection among high-risk people such as elderly and immuno-compromised individuals manifests in massive airway inflammation, which leads to the development of pneumonia [4]–[6]. Furthermore, there is a constant threat from naturally evolving IAV strains in avian and animal reservoirs that can lead to an epidemic or pandemic. Death of more than 200,000 individuals due to swine IAV (2009 H1N1 IAV) associated infection [7] is an example of the catastrophic nature of IAV infection.

Innate immunity, comprised of antiviral activity (via type-I interferons, IFN-α/β) and a controlled inflammatory response, is critical host defense machinery for virus clearance and the resolution of virus-induced disease [8]–[16]. PRRs recognize PAMPs to induce innate immunity in response to pathogen invasion. During IAV infection, both membrane-bound (e.g., TLRs) and cytosolic (e.g., RIG-like receptors such as RIG-I and Nod-like receptors such as NLRP3 and Nod2) PRRs are required to launch an effective innate response [13], [17]–[31].

Activation of PRRs could serve as a double-edged sword: While operating as host defense factors, activated PRRs can also contribute to the progression of virus-induced disease. For example, although TLR4 is activated during IAV infection, studies with TLR4 KO mice have shown that TLR4 contributes to exacerbated lung disease and mortality in IAV-infected animals [22], [23]. Since pneumonia is an inflammatory disease [6], [32], it is imperative to characterize the molecular mechanisms and cellular factors responsible for uncontrolled inflammation mediated by TLR4 during IAV infection [23]. Although activated TLR4 is a key contributor to exacerbation of disease, the mechanism by which TLR4 is activated in IAV-infected cells is unknown, especially since IAV does not have TLR4-specific PAMP ligand lipopolysaccaride (LPS). Therefore, it is crucial to identify and characterize “non-PAMP” host-derived molecular pattern, which can activate PRRs during virus infection. We expect that this line of investigation will illuminate the role of host-factors in contributing, either positively or negatively, to IAV-associated disease and pathogenesis. These studies will be a stepping stone for development of therapeutics to combat IAV-associated lung disease.

We are interested in understanding the role of secreted soluble factors (e.g. defensins, interferon-alpha induced soluble factor) in viral innate immunity [15], [21], [33], [34]. During our studies to further understand how TLR response modulates expression/production of soluble secreted factors during infection, we found that cells lacking TLR adaptor TRIF failed to release S100A9 following IAV infection. We specifically focused on S100 proteins, since expression of both defensins and S100 proteins are concurrently enhanced during various cellular stimuli [35]–[37] and S100 proteins (S100A9 and S100A8) has implicated in activation of TLR4 pathway during LPS stimulation [38].

In the current study, we have identified extracellular S100A9 protein as a host-derived molecular pattern regulating the pro-inflammatory response, cell death, and pathogenesis during IAV infection. We also show that DDX21/TRIF and TLR4/MyD88 pathways are respectively required for S100A9 gene expression and activity. In addition, we have uncovered DDX21-TRIF-S100A9-TLR4-MyD88 signaling network as a critical regulator of inflammation. This network may also contribute to inflammation and disease during both infection-associated and noninfectious inflammatory diseases and disorders.

## Results

### S100A9 secretion from IAV-infected macrophages

**Figure 1:**
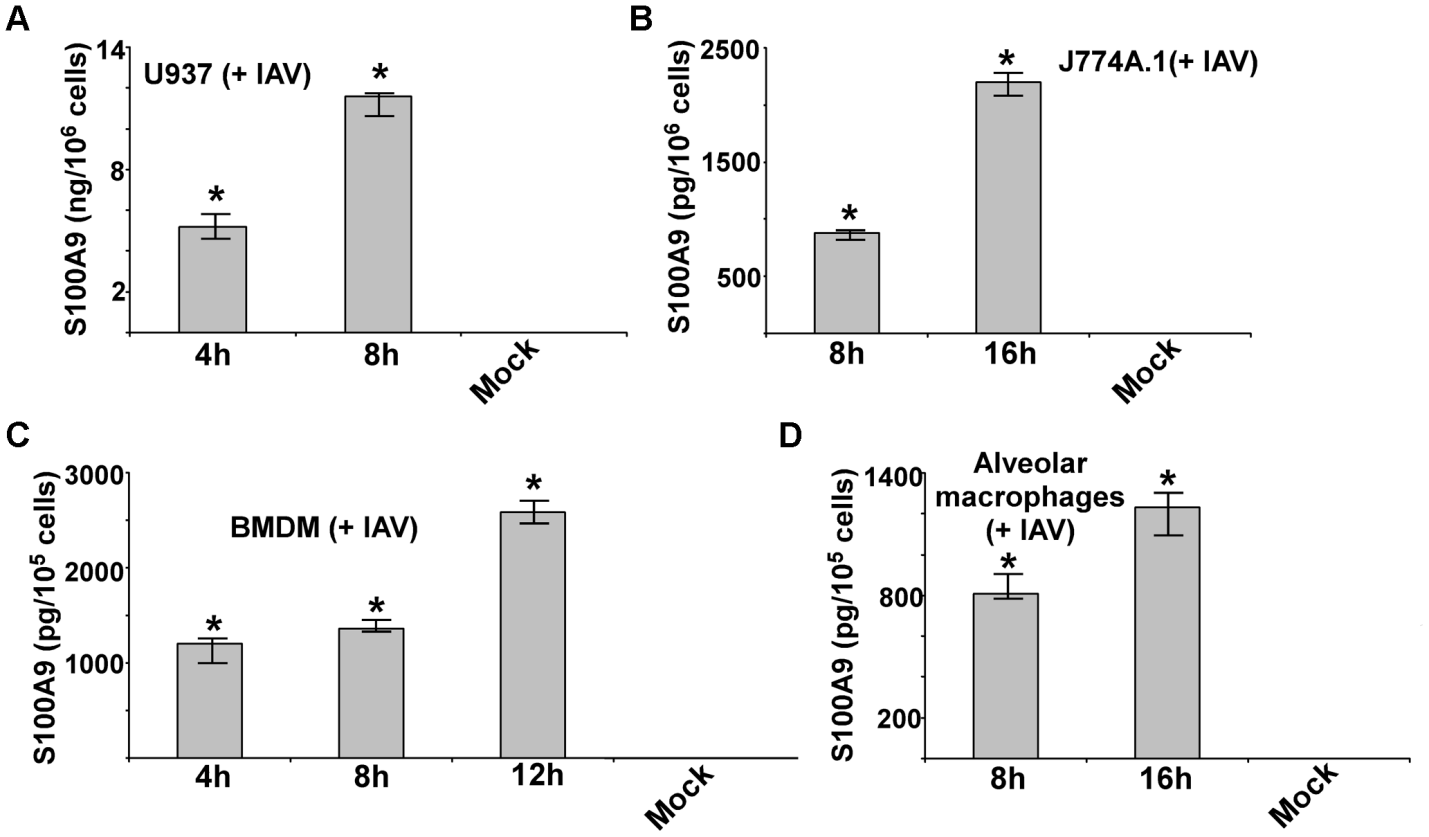
Production of S100A9 from IAV infected macrophages. U937 cells (**A**), J774A.1 cells (**B**), primary bone marrow derived macrophages or BMDM (**C**) and primary mouse alveolar macrophages (**D**) were infected with IAV. U937 cells were infected at 1 MOI, whereas J774A.1, BMDM and primary alvelolar macrophages were infected at 2 MOI. At indicated post-infection time-periods the medium supernatant was collected to assess levels of S100A9 protein by ELISA. The values shown represent the mean ± standard deviation from three independent experiments performed in triplicate. *p<0.05 using a Student's t test.

Macrophages are essential immune cells that modulate host defense, inflammation, and disease pathogenesis during IAV infection. Macrophages are also the major cytokine- and chemokine-producing cells during IAV infection and thus contribute to lung tissue damage [39]–[42]. To investigate whether IAV infection stimulates S100A9 secretion, we infected macrophages with IAV for 4–16 h. After infection, medium supernatant was collected to assess S100A9 protein levels by ELISA. We found that following IAV infection both human (U937 cells) (Figure 1A) and mouse [J774A.1 macrophage cell-line, primary alveolar macrophages and primary bone marrow-derived macrophages (BMDMs)] macrophages (Figure 1B–D) secreted high levels of S100A9. The physiological significance is evident from the ability of primary macrophages (i.e. alveolar macrophages and BMDMs) (Figure 1C and D) to secrete S100A9 upon IAV infection. Interestingly, S100A9 secretion was detected as early as 4–8 h postinfection. Release of S100A9 is not due to cell cytotoxicity or damage, since an LDH release cytotoxicity assay showed minimal cytotoxicity in macrophages at 8 and 16 h postinfection (Figure S1A). Similarly, no cell death (apoptosis or necrosis) was observed during the 8–16 h postinfection period (not shown). These results demonstrated that following IAV infection, S100A9 is released to the extracellular environment from undamaged macrophages.

### The DDX21/TRIF pathway is required for S100A9 gene expression and secretion during IAV infection

**Figure 2:**
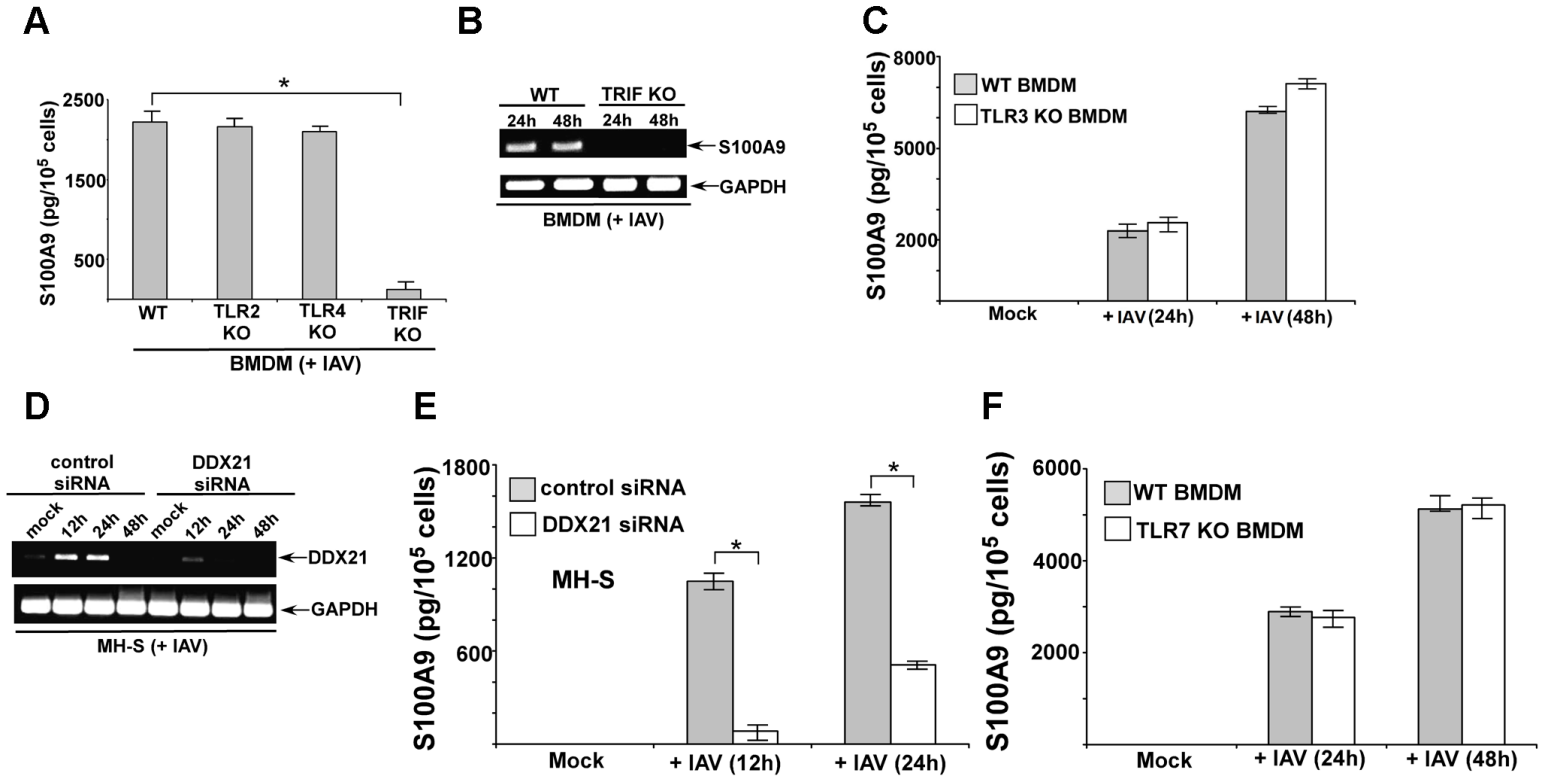
DDX21 and TRIF is required for S100A9 production from IAV infected macrophages. (**A**) Primary bone marrow derived macrophages or BMDMs isolated from wild-type (WT), TLR2 knockout (KO), TLR4 KO and TRIF KO mice were infected with IAV (2 MOI). At 24 h post-infection time-period the medium supernatant was collected to assess levels of S100A9 protein by ELISA. (**B**) RT-PCR analysis of S100A9 expression in IAV infected WT and TRIF KO BMDMs. (**C**) BMDMs isolated from WT and TLR3 KO mice were infected with IAV (2 MOI). At indicated post-infection time-periods the medium supernatant was collected to assess levels of S100A9 protein by ELISA. (**D**) Mouse alveolar macrophage cell-line MH-S was transfected with either control siRNA or DDX21 specific siRNA. At 48 h post-transfection, cells were infected with IAV (2 MOI). At indicated post-infection time-period RT-PCR analysis was performed to examine expression of DDX21 in IAV infected control and DDX21 silenced cells. (**E**) MH-S cells transfected with either control siRNA or DDX21 specific siRNA were infected with IAV (2 MOI). At indicated post-infection time-period the medium supernatant was collected to assess levels of S100A9 protein by ELISA. (**F**) BMDMs isolated from WT and TLR7 KO mice were infected with IAV (2 MOI). At indicated post-infection time-periods the medium supernatant was collected to assess levels of S100A9 protein by ELISA. The values shown in (A), (C), (E) and (F) represent the mean ± standard deviation from three independent experiments performed in triplicate. *p<0.05 using a Student's t test. Each RT-PCR data (B and D) is a representative of three independent experiments with similar results.

There have been no studies to date on the signaling mechanism that regulates gene expression of S100 family of proteins. We examined the signaling mechanism involved in S100A9 expression. We infected BMDMs derived from wild-type (WT), TLR2 knockout (KO), TLR4 KO and TRIF KO mice with IAV. At 24 h postinfection, we evaluated S100A9 levels in the medium. TLR2 and TLR4 were not involved, since comparable S100A9 secretion was noted in WT and TLR KO BMDMs (Figure 2A). A similar result was obtained with TRAM KO and TIRAP KO cells (not shown). In contrast, significant reduction in S100A9 secretion was observed in IAV-infected TRIF KO BMDMs (Figure 2A). RT-PCR analysis showed that loss of S100A9 secretion was caused by the absence of S100A9 mRNA in infected TRIF KO cells (Figure 2B). Apart from TLR4, which uses TRIF for MyD88-independent signaling, TLR3 also recruits TRIF for TLR3-mediated signal transduction. However, TLR3 is not involved in this process, as shown by the fact that S100A9 secretion was not reduced in TLR3 KO BMDMs (Figure 2C). These results demonstrated that S100A9 gene induction occurs via the TLR-independent TRIF-dependent pathway.

Recently, DEAD box proteins (also known as DDX protein) having RNA helicase activity has been implicated in innate immunity [43]. DDX proteins (e.g. DDX21) can function as cytosolic PRR in mouse dendritic cells (mDCs) to induce type-I interferon during infection [43]. Interestingly, DDX signaling was TRIF-dependent and DDX21 interacted with TRIF during signaling [43]. Therefore, we examined whether DDX21 has a role in S100A9 expression during IAV infection of macrophages. Since KO animals lacking DDX proteins do not exist, we used siRNA technology to silence DDX21 expression in macrophages.

Mouse alveolar macrophages (MH-S cell line) were transfected with DDX21-specific siRNA or control scrambled siRNA, after which these cells were infected with IAV. DDX21 expression was monitored by RT-PCR. We observed induction of DDX21 expression following IAV infection (Figure 2D). The silencing efficiency was evident from the loss of DDX21 expression in IAV-infected cells transfected with DDX21-specific siRNA (Figure 2D). We used the silenced cells to deduce the role of DDX21 in S100A9 gene expression following IAV infection. DDX21 is critical for S100A9 gene expression, since drastic loss of S100A9 mRNA was observed in IAV-infected DDX21 silenced cells (Figure S1B). Accordingly, reduced S100A9 mRNA expression in DDX21 silenced cells led to diminished S100A9 secretion following IAV infection of these cells (Figure 2E). The DDX/TRIF dependent S100A9 expression was independent of virus replication, since IAV hemagglutinin (HA) mRNA levels were similar in DDX21 silenced and TRIF KO cells (Figure S2A and S2B). Moreover, S100A9 expression (not shown) and production (Figure S2C and S2D) was not significantly altered in IAV infected MyD88 KO and MAVS KO cells, which implicated MyD88 aααnd MAVS independent expression/production of S100A9 during IAV infection. In addition, we failed to observe significant difference in S100A9 expression/production from IAV infected WT vs. TLR7 KO cells (Figure 2F). It is interesting to note that DDX21 expression was undetected at 48 h postinfection (Figure 2D), which co-relates with loss of S100A9 production during that time frame (not shown). This suggests that to maintain homeostasis and to avoid hyper-inflammation cells may negatively regulate DDX21 expression to reduce S100A9 production. These results demonstrated that the DDX21/TRIF pathway is required for S100A9 gene induction and the resulting S100A9 secretion following IAV infection.

### Extracellular S100A9 promotes pro-inflammatory response during IAV infection

In the preceding studies, the high levels of S100A9 secretion during infection suggested that secreted extracellular S100A9 may have some role during IAV infection. Therefore, we focused on the role and function of extracellular S100A9 during IAV infection. Earlier studies have shown that the S100A9/S100A8 complex is required for optimal LPS-induced TLR4-dependent TNF-α (TNF) production in bone marrow cells (comprised of undifferentiated monocytes and DCs) [38]. However, few studies have shown the pro-inflammatory activity of S100A9 in the absence of S100A8 and LPS. Moreover, it is not known whether S100A9 can launch a pro-inflammatory response in macrophages. Since our studies are focused on the innate responses of IAV-infected macrophages, we investigated whether extracellular addition of purified S100A9 protein (to mimic secreted S100A9) promotes the release of pro-inflammatory cytokines IL-6 and TNF-α (TNF). These pro-inflammatory cytokines are produced early during IAV infection, a period that corresponds with S100A9 secretion kinetics.

**Figure 3:**
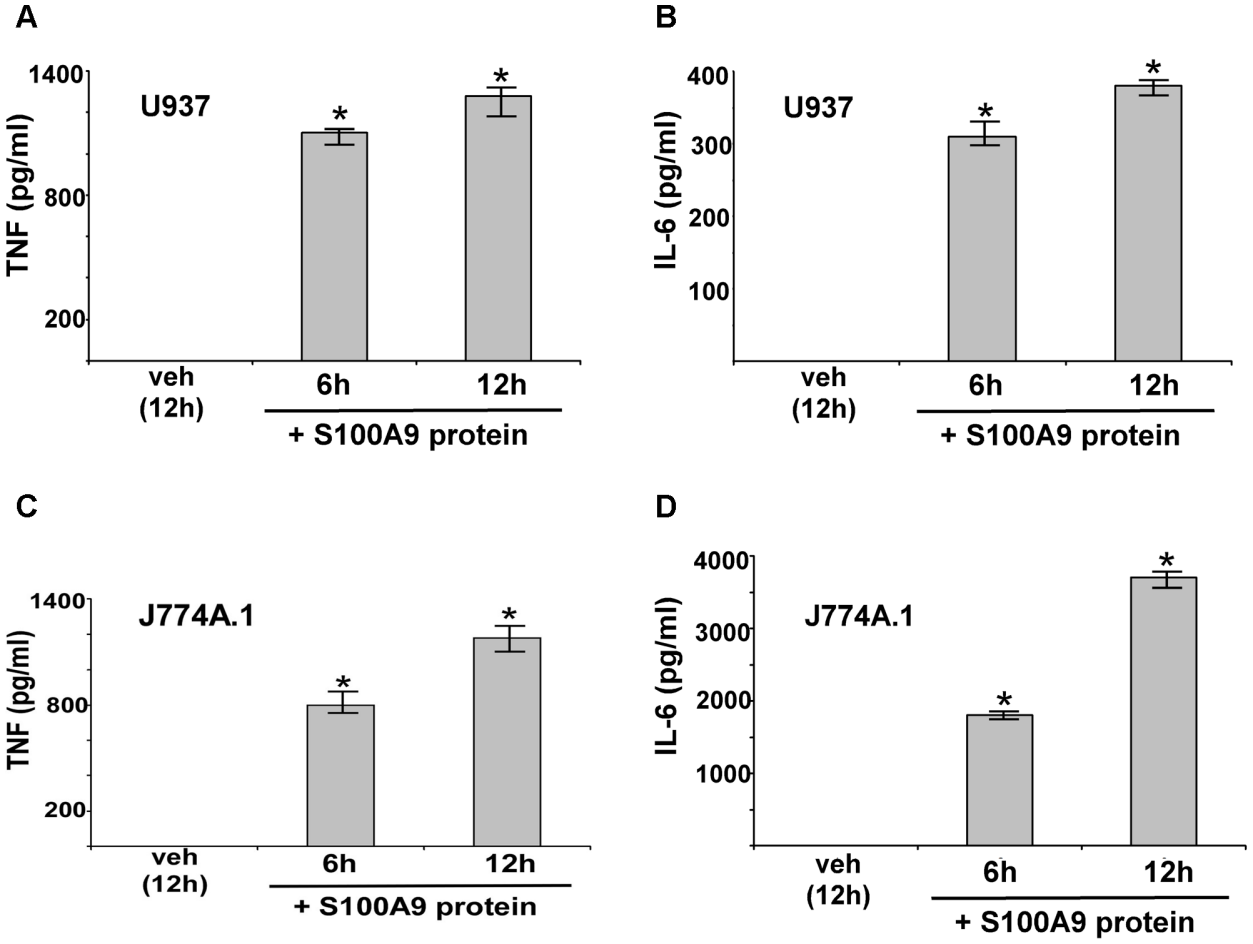
Extracellular S100A9 protein stimulates pro-inflammatory response in macrophages. Human U937 macrophages were incubated with purified recombinant human S100A9 protein (10 µg/ml) for 6 h and 12 h. The medium supernatant was collected to assess levels of human TNF-α (TNF) (**A**) and human IL-6 (**B**) by ELISA. Mouse J774A.1 macrophages were incubated with purified recombinant mouse S100A9 protein (5 µg/ml) for 6 h and 12 h. The medium supernatant was collected to assess levels of mouse TNF (**C**) and mouse IL-6 (**D**) by ELISA. The values represent the mean ± standard deviation from three independent experiments performed in triplicate. *p<0.05 using a Student's t test. Vehicle control cells (veh) were incubated with HBSS buffer.

Mouse (J774A.1) and human (U937 cells) macrophages were incubated with purified mouse or human S100A9 proteins, respectively for 6–12 h (Figure 3). After treatment, medium supernatant was collected to analyze TNF and IL-6 levels by ELISA. S100A9 alone stimulates a pro-inflammatory response in macrophages, as is evident from high levels of TNF (Figure 3A and C) and IL-6 (Figure 3B and D) production by macrophages treated with purified S100A9 protein. Both human (Figure 3A and B) and mouse (Figure 3C and D) macrophages produced pro-inflammatory cytokines upon incubation with human and mouse S100A9 protein. Interestingly, the response was rapid, since substantial TNF and IL-6 production occurred within 6 h of treatment with S100A9 protein. RT-PCR analysis showed that production of TNF and IL-6 by S100A9 was due to activation of their corresponding genes (Figure S3). Since the pro-inflammatory activity of purified S100A9 protein could be inhibited by anti-S100A9 blocking (neutralizing) antibody (not shown), the observed response was due to S100A9 protein. Moreover, the effect observed with purified S100A9 protein was not due to LPS contamination (Figure S4A and S4B). These studies demonstrated that S100A9 functions as an extracellular host factor to launch a pro-inflammatory response in macrophages.

**Figure 4:**
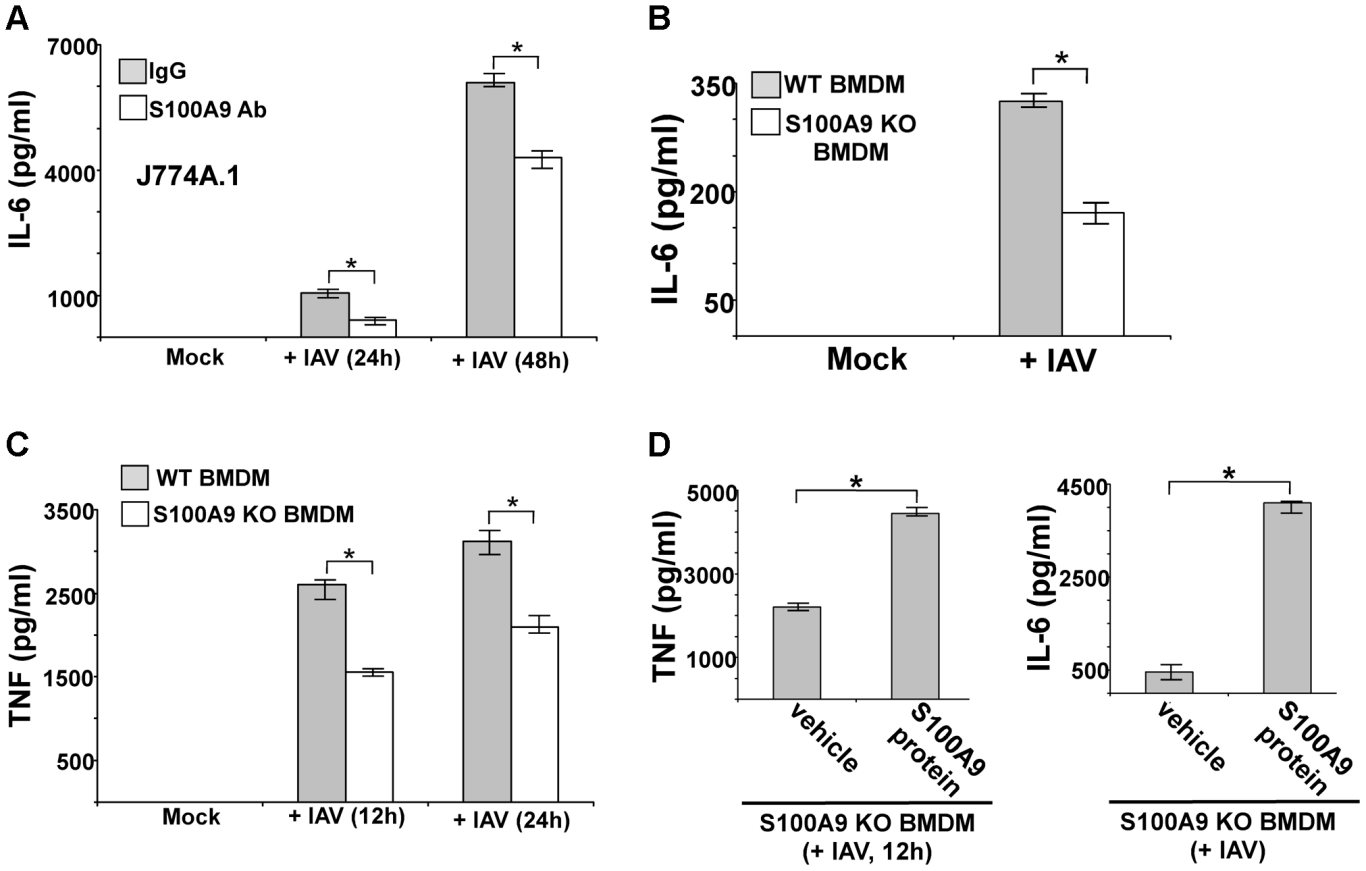
Extracellular S100A9 plays an essential role in inducing pro-inflammatory response during IAV infection of macrophages. (A) Mouse J774A.1 macrophages were infected with IAV (2 MOI) in the presence of either control IgG (IgG) or anti-S100A9 blocking (neutralizing) antibody (S100A9 Ab). At indicated post-infection time-periods the medium supernatant was collected to assess levels of mouse IL-6 by ELISA. (**B**) Primary bone marrow derived macrophages (BMDM) isolated from wild-type (WT) and S100A9 knockout (KO) mice were infected with IAV (2 MOI). The medium supernatant was collected to assess levels of mouse IL-6 by ELISA. (**C**) WT and S100A9 KO BMDM were infected with IAV (2 MOI). At the indicated post-infection time-period, medium supernatant was collected to assess levels of mouse TNF-α(TNF) by ELISA. (**D**) S100A9 KO BMDMs were infected with IAV (2 MOI) in the presence of purified recombinant mouse S100A9 protein (5 µg/ml). Medium supernatant was collected from infected cells to assess levels of mouse TNF and IL-6 by ELISA. Vehicle control cells (veh) were incubated with HBSS buffer. The values represent the mean ± standard deviation from three independent experiments performed in triplicate. *p<0.05 using a Student's t test.

We next examined the role of secreted S100A9 in eliciting a pro-inflammatory response during IAV infection. We used blocking antibody against S100A9 to neutralize the activity of extracellular (secreted) S100A9. Previously, it was shown that this blocking antibody specifically inhibited the activity of the secreted extracellular form of S100A9 both *in vitro* and *in vivo* [44]–[50]. J774A.1 cells were infected with IAV in the presence of either control antibody (control IgG) or S100A9-specific blocking antibody. At various postinfection time points, IL-6 and TNF levels were examined by ELISA. Extracellular S100A9 plays a key role in inducing the pro-inflammatory response during IAV infection, since significant reduction in IL-6 (Figure 4A) and TNF (Figure S4C) levels were observed in infected cells treated with S100A9 blocking antibody. RT-PCR showed that loss of IL-6 and TNF production was due to diminished gene expression (not shown). Similar results were obtained following treatment of IAV-infected primary macrophages (BMDM) with S100A9 blocking antibody (Figure S4D and S4E). Diminished IL-6 (Figure S4D and S4E) and TNF (not shown) production (Figure S4D) and expression (Figure S4E) was observed in infected BMDM treated with S100A9 blocking antibody. The loss of pro-inflammatory response was not due to reduced IAV replication, since IAV HA expression was similar in control antibody and S100A9 blocking antibody treated J774A.1 cells (Figure S5A) and BMDMs (Figure S5B). Thus, extracellular S100A9 modulates pro-inflammatory response independent of IAV replication.

Our finding that S100A9 contributes to the pro-inflammatory response during IAV infection was validated by using BMDMs derived from S100A9 KO mice. WT and S100A9 KO BMDMs were infected with IAV, after which TNF and IL-6 levels in the medium supernatant were measured by ELISA. As compared to WT cells, there were significant reductions in IL-6 (Figure 4B) and TNF (Figure 4C) production from infected S100A9 KO cells. Once again, this was a consequence of the loss of pro-inflammatory gene expression in IAV-infected S100A9 KO BMDMs (Figure S5C). The critical function of secreted (extracellular-form) S100A9 during this response was apparent from the observation that addition of purified mouse S100A9 protein to S100A9 KO BMDMs restored the pro-inflammatory response in IAV-infected S100A9 KO BMDMs (Figure 4D). This result also suggested that intracellular S100A9 does not play a role in inducing a pro-inflammatory response. Treatment of WT or S100A9 BMDMs with S100A9 protein did not alter IAV replication status in the corresponding cells (not shown). We also observed production of S100A9 following treatment of BMDMs with synthetic dsRNA (poly-IC) (Figure S6A). The pro-inflammatory activity of S100A9 was specific for IAV and dsRNA (which serves as a replicative intermediate during IAV infection and induces DDX21/TRIF pathway) since dsRNA (poly-IC) dependent TNF and IL-6 release was significantly diminished in S100A9 KO cells (Figure S6B and S6C), and treatment of KO cells with purified S100A9 protein restored the pro-inflammatory response in poly-IC treated S100A9 KO cells (Figure S6D and S6E). In contrast, TNF and IL-6 release from S100A9 KO BMDMs was not affected following imiquimod (which activates TLR7 dependent pro-inflammatory response) treatment (Figure S6F and S6G). In addition, treatment of WT and S100A9 KO BMDMs with TNF (to induce NF-κB dependent inflammatory response via TNF receptor) revealed similar levels of IL-6 production from both WT and KO cells (Fig. S6H).

During these studies we observed that IAV replication (as deduced from IAV HA mRNA expression) was significantly reduced in S100A9 KO BMDMs compared to WT cells (Figure S7A). This result suggested that although extracellular S100A9 plays a critical role in modulating pro-inflammatory response (Figure 4D), intracellular S100A9 may be involved in negatively regulating antiviral factor expression/production or it is required for efficient virus infection/replication. This is not surprising in light of previous reports illustrating differential function of extracellular vs. intracellular S100 proteins.

It is important to mention that we observed S100A9 production from IAV-infected BMDMs at 4 h postinfection (Figure 1C) and that TNF and IL-6 are produced from IAV-infected BMDMs at 8–12 h postinfection (not shown); these cytokines are undetectable at 4 h postinfection (not shown). Thus, S100A9 secretion and production of early pro-inflammatory mediators (e.g. TNF, IL-6) are temporally regulated during IAV-infection. Therefore, extracellular S100A9 is a key regulator of the pro-inflammatory response during IAV infection.

### Extracellular S100A9 promotes apoptosis during IAV infection

Macrophages undergo apoptosis during IAV infection [41], [42]. Several studies have demonstrated that S100A9 has a pro-apoptotic function in epithelial cells, muscle cells, and neutrophils [51]–[55], but no apoptosis-inducing activity of S100A9 (or any other S100 proteins) in macrophages has been reported. Since IAV infection resulted in high levels of S100A9 secretion, we examined the ability of extracellular S100A9 to induce apoptosis in macrophages and the role of secreted S100A9 in apoptotic induction during IAV infection.

**Figure 5:**
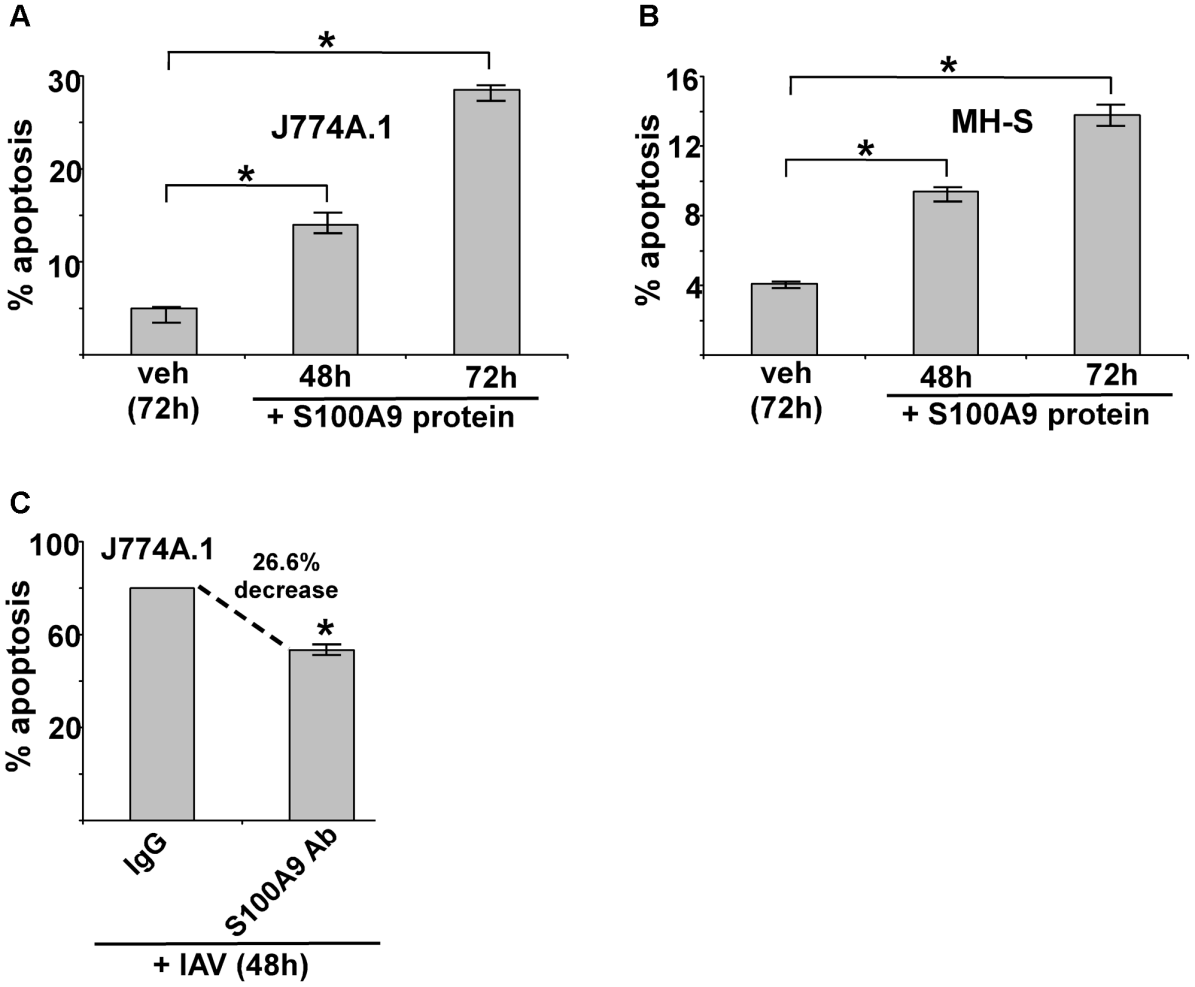
Extracellular S100A9 protein triggers apoptosis in macrophages and S100A9 regulates apoptosis during IAV infection. (**A**) Mouse J774A.1 macrophages were incubated with purified recombinant mouse S100A9 protein (5 µg/ml) for 48 h and 72 h. The apoptotic state of these cells was examined by FACS analysis of annexin V and PI stained cells. Apoptosis rate (% apoptosis) was calculated based on number of annexin V positive/PI negative cells (denoting early apoptosis)+number of annexin V positive/PI positive cells (denoting late apoptosis)/total number of cells. (**B**) Mouse alveolar macrophage MH-S cell-line was incubated with purified S100A9 protein (5 µg/ml) for 48 h and 72 h. The apoptotic status was determined as described in (A). (**C**) Mouse J774A.1 macrophages were infected with IAV (2 MOI) in the presence of either control IgG (IgG) or anti-S100A9 blocking (neutralizing) antibody (S100A9 Ab). At 48 h post-infection, the apoptotic state of these cells was determined as described in (A). The values (i.e. annexin V and PI staining quantified by FACS) represents mean ± standard deviation from three independent experiments, *p<0.05 by Student's t test. Veh; cells incubated with HBSS buffer (vehicle control).

We treated J774A.1 and MH-S macrophages with purified S100A9 protein for 48 and 72 h, then examined the apoptotic status of cells by monitoring annexin V and PI staining. The apoptosis rate was calculated based on the number of annexin V positive/PI negative cells (denoting early apoptosis)+number of annexin V positive/PI-positive cells (denoting late apoptosis) per total number of cells. We noted apoptosis in S100A9 protein-treated mouse macrophages (Figure 5A and B). The result obtained with annexin V and PI staining was confirmed by performing TUNEL analysis (Figure S7B and S7C). Similar results were obtained following treatment of human U937 macrophages (not shown).

Since IAV infection triggers S100A9 secretion, we next examined whether extracellular S100A9 has a role in apoptosis of IAV-infected macrophages. J774A.1 macrophages were infected with IAV for 48 h in the presence of either control antibody (control IgG) or S100A9 blocking antibody. Significantly diminished apoptosis (reduction of 27%) occurred in macrophages treated with S100A9 antibody (Figure 5C). These results were further confirmed by TUNEL analysis (Figure S7D). Thus, extracellular S100A9 has a critical function in regulating apoptosis of IAV-infected macrophages.

### The TLR4/MyD88 pathway is required for S100A9-mediated pro-inflammatory response following IAV infection

**Figure 6:**
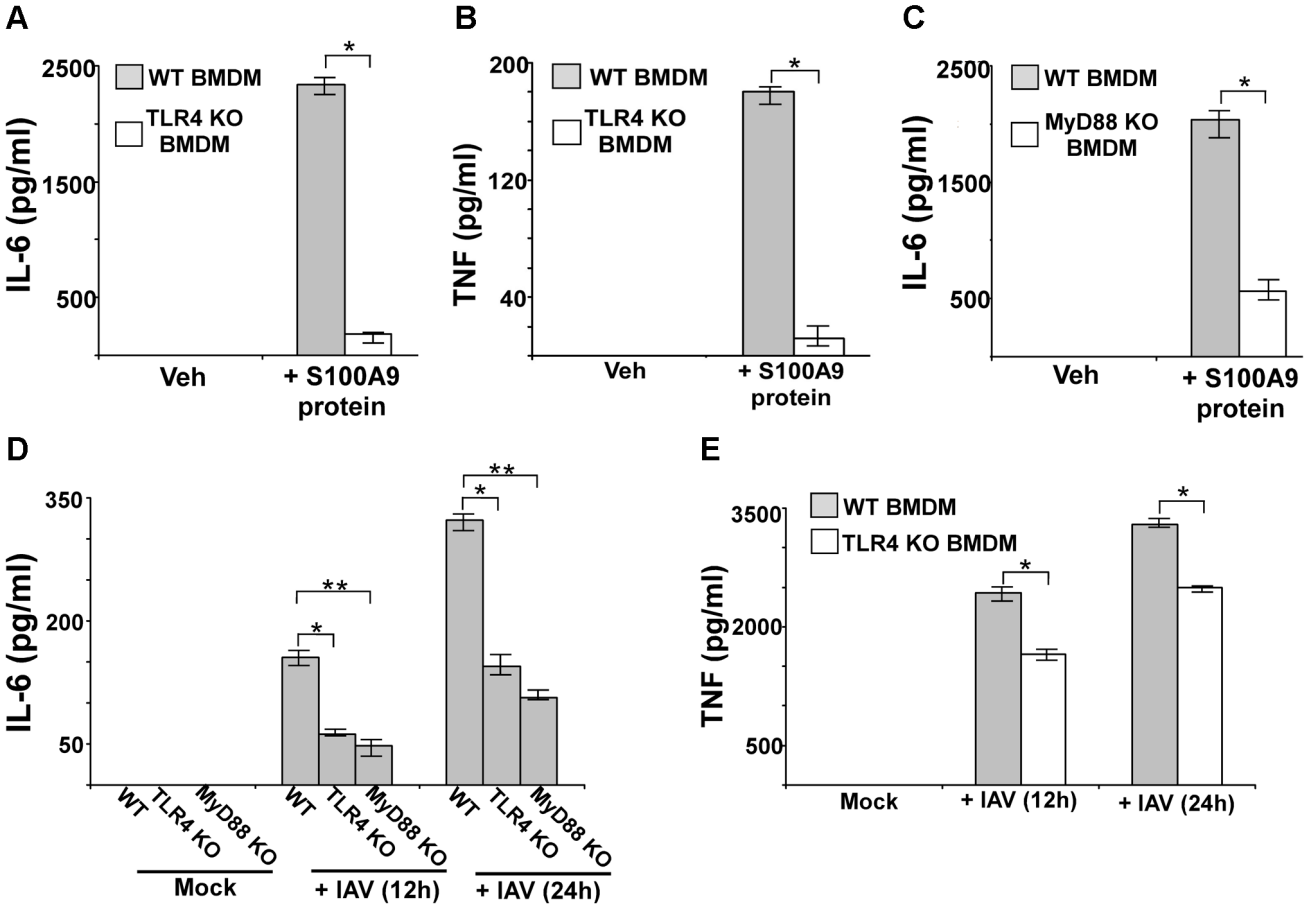
S100A9 activates TLR4/MyD88 pathway and activation of TLR4/MyD88 pathway is essential for IAV-induced pro-inflammatory response. Primary bone marrow derived macrophages (BMDM) isolated from wild-type (WT) and TLR4 knockout (KO) mice were incubated with purified recombinant mouse S100A9 protein (5 ug/mL). The medium supernatant was collected to assess levels of mouse IL-6 (**A**) and mouse TNF-α(TNF) (**B**) by ELISA. (**C**) IL-6 production from S100A9 protein treated WT and MyD88 KO BMDMs. (**D**) BMDM isolated from WT, TLR4 KO and MyD88 KO mice were infected with IAV (2 MOI). At 12 h and 24 h post-infection time-period, medium supernatant was collected to assess levels of mouse IL-6 by ELISA. (**E**) TNF production from IAV infected WT and TLR4 KO BMDMs. The values represent the mean ± standard deviation from three independent experiments performed in triplicate, *p<0.05 using a Student's t test. Veh; cells incubated with HBSS buffer (vehicle control).

Previous studies have found that optimal TLR4 activation by LPS in bone-marrow cells required the activity of extracellular S100A9/S100A8 complex [38]. However, it is not known whether S100A9 alone activates TLR4, especially in macrophages. In addition, there has been no report of DAMP proteins like S100A9 activating PRR signaling during virus infection. Therefore, we investigated the role of the TLR4/MyD88 pathway in the macrophage pro-inflammatory response by S100A9 alone (in the absence of S100A8), and the function of the S100A9/TLR4/MyD88 pathway in regulating the pro-inflammatory response in IAV-infected macrophages. We incubated WT and TLR4 KO BMDMs with purified S100A9 protein, and then measured IL-6 (Figure 6A) and TNF (Figure 6B) levels by ELISA. Drastic loss of IL-6 and TNF production was detected in S100A9 protein-treated TLR4 KO BMDMs (Figure 6A and B), indicating that TLR4 is absolutely required for the S100A9-mediated response. Drastic reductions in IL-6 (not shown) and TNF (Figure S8A) transcripts occurred in S100A9 protein treated TLR4 KO cells.

Since MyD88 is one of the critical adaptors for activated TLR4, we next investigated the role of MyD88 by using MyD88 KO BMDMs. Incubation of WT and MyD88 KO BMDMs with purified S100A9 protein significantly reduced production of IL-6 (Figure 6C) and TNF (not shown) from MyD88 KO cells. The loss of cytokine protein production was due to reduced TNF (Figure S8B) and IL-6 (not shown) gene expression in MyD88 KO BMDMs, thus, demonstrating that the TLR4/MyD88 pathway is required for the S100A9-mediated pro-inflammatory response.

We next studied the role of TLR4/MyD88 in stimulating the pro-inflammatory response following IAV infection. After WT, MyD88 KO, and TLR4 KO BMDMs were infected with IAV, IL-6 levels were assessed by ELISA. Our study revealed that TLR4/MyD88 is an essential regulator of pro-inflammatory response during IAV infection, since significant reduction in IL-6 (Figure 6D) and TNF (Figure 6E) production was noted in IAV infected TLR4 KO (Figure 6D and E) and MyD88 KO (Figure 6D) BMDMs. RT-PCR analysis demonstrated diminished expression of IL-6 mRNA in TLR4 KO (Figure S8C) and MyD88 KO (not shown) BMDMs. Similarly, we noted significant reduction in TNF production from IAV-infected TLR4 KO (Figure 6E) and MyD88 KO (not shown) BMDMs. The observed effect was independent of virus replication since compared to WT cells, no change in HA mRNA expression was noted in TLR4 KO (Figure S8D) and MyD88 KO (not shown) cells. Thus, TLR4/MyD88 activation is a key step for inducing the S100A9-mediated pro-inflammatory response. Also, the S100A9/TLR4/MyD88 pathway is a crucial regulator of the pro-inflammatory response during IAV infection.

### TLR4/MyD88 pathway is required for apoptosis during IAV infection

**Figure 7:**
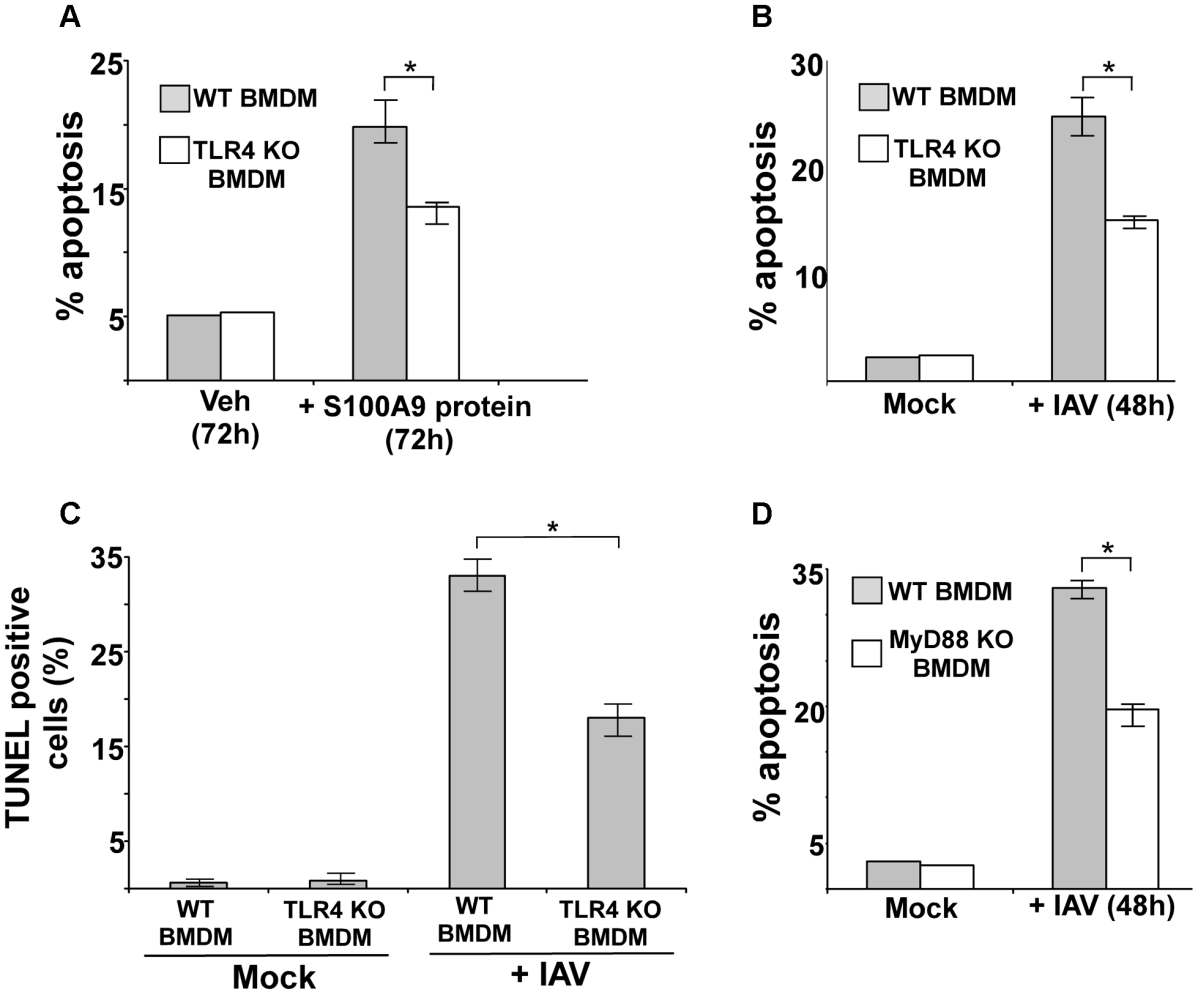
Activated TLR4/MyD88 pathway promotes S100A9-mediated apoptosis and is required for optimal apoptosis of IAV infected cells. (**A**) Primary bone marrow derived macrophages (BMDM) isolated from wild-type (WT) and TLR4 knockout (KO) mice were incubated with purified recombinant mouse S100A9 protein (5 ug/mL) for 72 h. The apoptotic state of these cells was examined by FACS analysis of annexin V and PI stained cells. Apoptosis rate (% apoptosis) was calculated based on number of annexin V positive/PI negative cells (denoting early apoptosis)+number of annexin V positive/PI positive cells (denoting late apoptosis)/total number of cells. (**B**) WT and TLR4 KO BMDMs were infected with IAV (1 MOI). At 48 h post-infection, the apoptotic status was determined as described in (A). (**C**) IAV infected WT and TLR4 KO cells were subjected to TUNEL assay. TUNEL positive cells were analyzed by image J software. Percent TUNEL positive cells denotes ratio of number of TUNEL positive cells/total number of cells. (**D**) WT and MyD88 KO BMDMs were infected with IAV (1 MOI). At 48 h post-infection, the apoptotic status was determined. The values represents mean ± standard deviation from three independent experiments, *p<0.05 by Student's t test. Veh; cells incubated with HBSS buffer (vehicle control).

Our study showed that extracellular S100A9 uses TLR4/MyD88 signaling for the pro-inflammatory response during IAV infection. TLR4 activation has been associated with apoptosis induction via various mechanisms, including activation of the pro-apoptotic function of NF-κB, modulation of tumor-suppresser expression or function etc [56]–[62]. To assess the role of TLR4 in S100A9-mediated apoptosis, we treated WT and TLR4 KO BMDMs with purified S100A9 protein for 72 h. Treatment of WT BMDMs with S100A9 protein induced apoptosis (Figure 7A), which was consistent with our previous findings. However, significant loss of apoptosis was observed in S100A9 protein-treated TLR4 KO BMDMs (Figure 7A).

We next examined the role of TLR4 and MyD88 in apoptosis induction during IAV infection. We infected WT and TLR4 KO BMDMs with IAV and evaluated apoptosis 48 h later. Apoptosis analysis by annexin V staining (Figure 7B) and TUNEL (Figure 7C) revealed that while IAV infection resulted in apoptosis of WT macrophages, a significant reduction in apoptosis was detected in TLR4 KO cells. Diminished apoptosis was also observed in infected MyD88 KO BMDMs (Figure 7D), indicating that MyD88 is also required during this event. Thus, the S100A9/TLR4/MyD88 pathway constitutes one of the mechanisms that modulate apoptosis of IAV-infected cells.

### S100A9 expression in IAV-infected mouse respiratory tract

**Figure 8:**
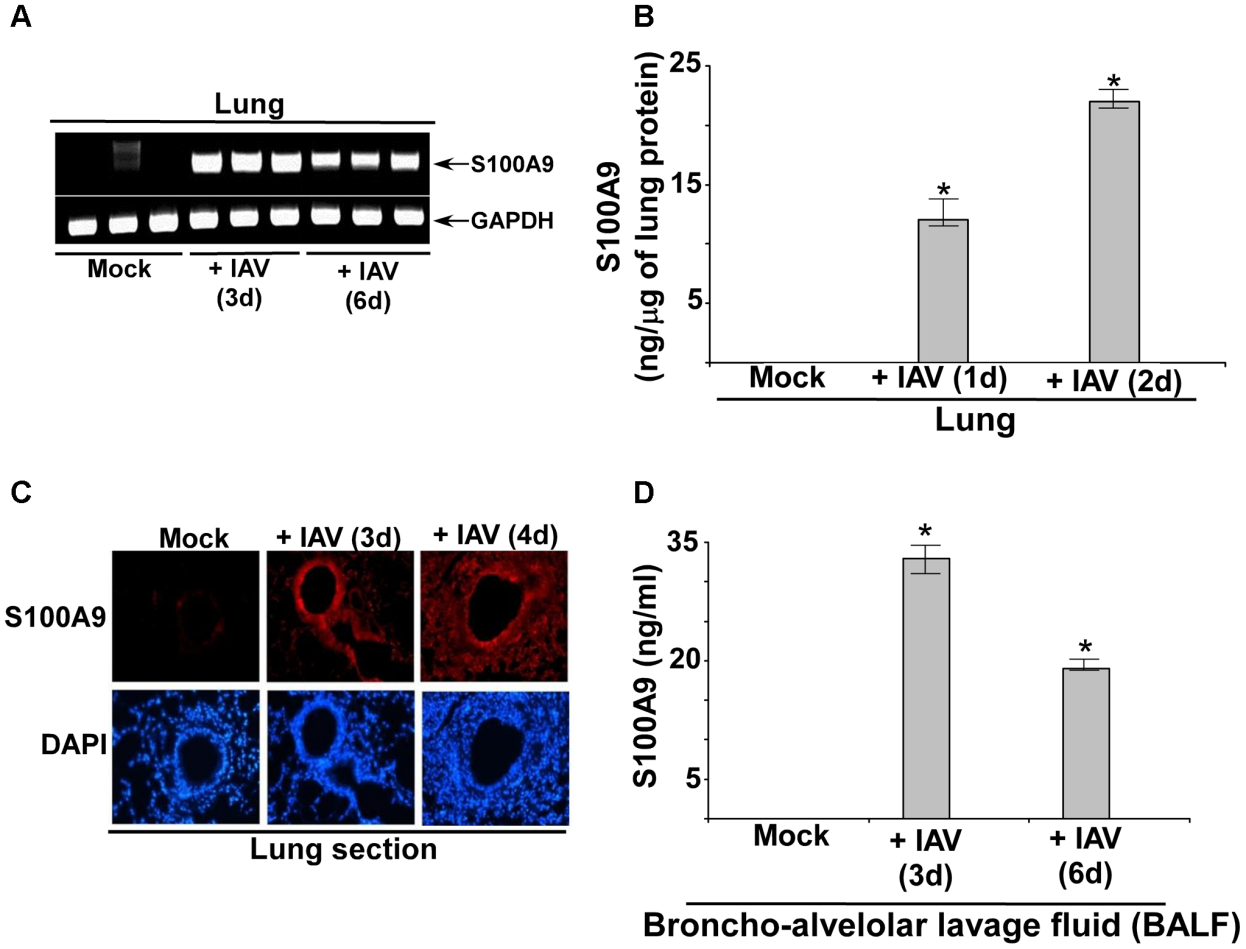
S100A9 expression and production in the IAV infected respiratory tract. (**A**) RNA isolated from mock infected and IAV infected (2×10^4^ pfu/mouse via intra-tracheal route) mice were subjected to RT-PCR analysis to examine expression of mouse S100A9. The RT-PCR data represents three mice/group (i.e. three mock mice, three mice infected with IAV for 3 d and three mice infected with IAV for 6 d). The RT-PCR data is a representative of three independent experiments with similar results. (**B**) Lung homogenate prepared from mock infected and IAV infected (2×10^4^ pfu/mouse via intra-tracheal route) mice were subjected to ELISA analysis to determine levels of mS100A9 protein in the lung. (**C**) Immuno-histochemical analysis of mouse lung tissue sections derived from mock infected and IAV infected mice were stained with mouse S100A9 antibody. Magnification, 200×. One representative example of a total of 3 mice analyzed per group in two independent experiments. (**D**) Broncho-alveolar lavage fluid (BALF) isolated from mock infected and IAV infected (2×10^4^ pfu/mouse via intra-tracheal route) mice were subjected to ELISA analysis to determine levels of S100A9 protein in BALF. The values shown in (B) and (D) represent the mean ± standard deviation from three independent experiments performed in triplicate. *p<0.05 using a Student's t test.

To establish the *in vivo* role of S100A9 in regulating innate response during IAV infection of the airway, we next evaluated S100A9 expression and its secretion in the IAV-infected mouse respiratory tract. Mice were intratracheally inoculated with IAV and, at 1–6 days postinfection, lungs were harvested. S100A9 mRNA expression in the lungs were analyzed by RT-PCR. S100A9 transcripts were observed in infected lungs but not in lungs from uninfected animals (Figure 8A), indicating that IAV infection led to robust induction of S100A9 gene expression. We also detected high levels of S100A9 protein in the lungs of IAV-infected mice (Figure 8B). Immunohistochemical analysis of lung sections confirmed the presence of S100A9 protein in IAV-infected animals (Figure 8C), while S100A9 was nearly undetectable in mock-infected lungs. Analysis of bronchoalveolar lavage fluid (BALF) by ELISA confirmed the presence of S100A9 protein in the airway of IAV-infected animals (Figure 8D). Thus, IAV infection of the respiratory tract results in induction of S100A9 gene expression and secretion of S100A9 protein in the airway.

### Extracellular S100A9 regulates IAV susceptibility and lung inflammation

Macrophages play a vital role in the innate response to IAV infection by producing pro-inflammatory mediators that determine the inflammation status in the lung [39]–[42]. Moreover, debris from dead cells, originating from apoptosis of immune cells, contributes to airway inflammation [40]–[42], [63]–[68]. Since extracellular S100A9 acted as a positive regulator of pro-inflammatory response and induced apoptosis, we hypothesized that extracellular S100A9 exacerbates IAV-associated lung disease. To test this, we used anti-S100A9 blocking antibody, which neutralizes extracellular (secreted) S100A9 protein.

We used anti-S100A9 antibody instead of doing our *in vivo* studies with S100A9 KO mice because S100 proteins have both intracellular functions (such as cytoskeletal rearrangement, cell metabolism, intracellular calcium response etc) and extracellular functions [69], [70]. Since we have elucidated a role of extracellular (secreted form) S100A9, results from KO mice might not distinguish whether the observed effects are due to activity of extracellular S100A9 or intracellular S100A9 function. Most importantly our studies demonstrated that intracellular S100A9 could be involved in negatively regulating antiviral response or it is required for IAV infection/replication, since reduced virus replication was noted in S100A9 KO BMDM compared to WT cells (Figure S7A). In that scenario, S100A9 KO mice may not serve as an appropriate model to study IAV-induced pro-inflammatory (and apoptotic) response *in vivo*, since viral burden in the lung is directly proportional to the degree of pro-inflammatory (and apoptotic) response (i.e. if there is less viral burden then concomitantly reduced pro-inflammatory response and apoptosis will occur). However, neutralizing the activity of extracellular S100A9 with S100A9 blocking antibody did not alter IAV replication *in vitro* (Figure S5A and S5B) and *in vivo* (please see below). We therefore used anti-S100A9 blocking antibody to specifically inhibit the activity of extracellular S100A9 in mice. We have previously shown that anti-S100A9 blocking antibody has extracellular S100A9 blocking activity [44]–[50]. Specifically, intraperitoneal (i.p) injection of S100A9 blocking antibody inhibited the activity of mouse S100A9 during *S. pneumoniae* infection [49]. Thus, this antibody [44], [45], [48]–[50] is useful to assess the functional role of extracellular (secreted form) S100A9. Furthermore, similar levels of S100A9 protein were detected in the BALF of control IgG-treated and S100A9-antibody treated mice (Figure S9A). Thus, i.p.-injected anti-S100A9 antibody did not significantly affect S100A9 protein production in the airway-lumen following IAV infection. As in previous reports, we detected anti-S100A9 antibody (administered i.p.) in lung homogenate (Figure S9B). Thus, anti-S100A9 antibody could effectively block lung-localized S100A9 during IAV infection [49], [71], [72]. The clinical significance of utilizing neutralizing antibody is obvious from possible passive immunization with S100A9 antibody as a new therapeutic strategy to control lung inflammation and associated lung disease during IAV infection.

**Figure 9:**
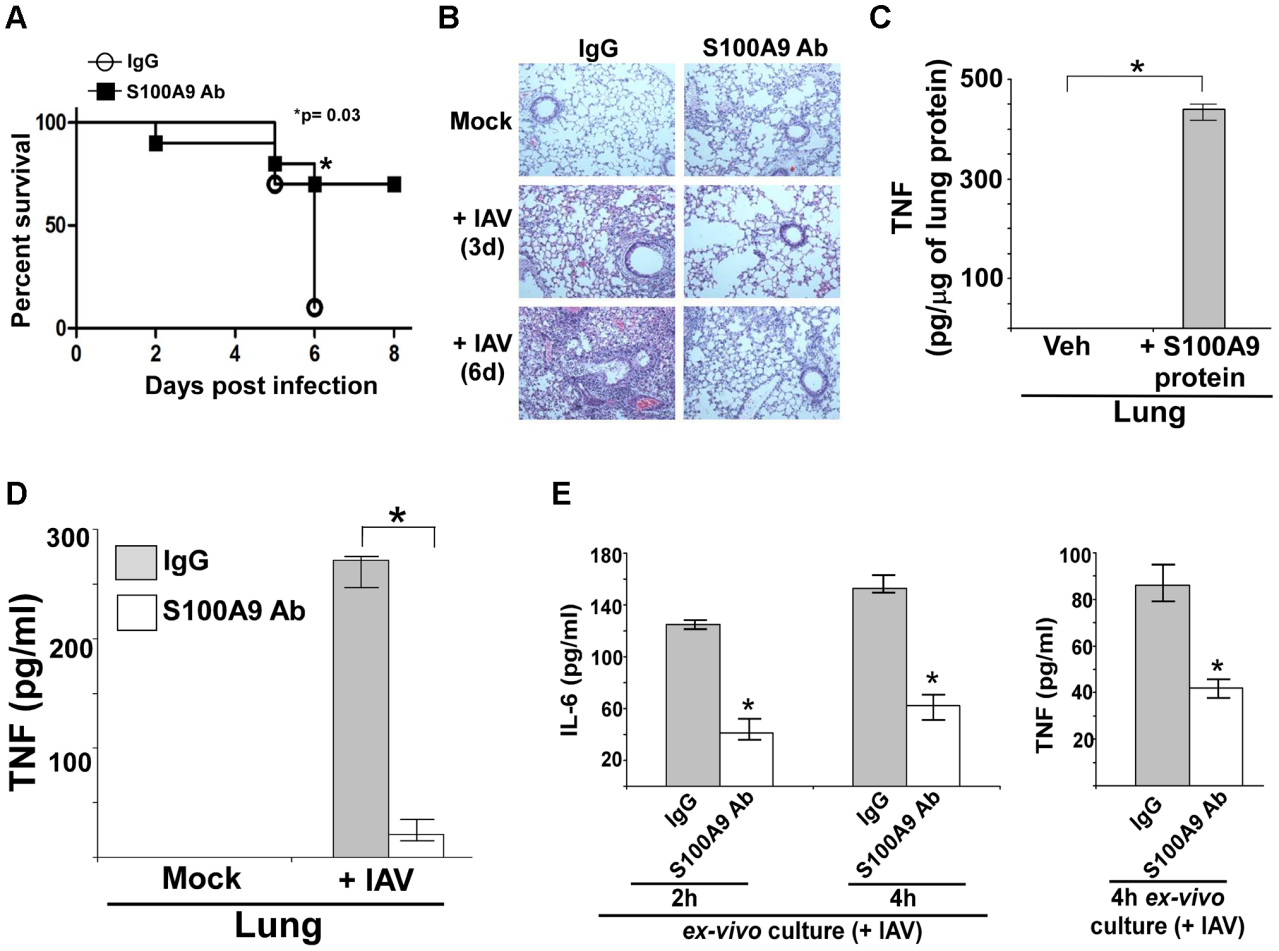
S100A9 contributes to enhanced susceptibility and inflammation during IAV infection and S100A9 regulates pro-inflammatory response in the respiratory tract of IAV infected mice. (**A**) Survival of IAV infected (1×10^5^ pfu/mouse via intra-tracheal route) mice administered with either control IgG (IgG) or anti-S100A9 blocking (neutralizing) antibody (S100A9 Ab) (24 h prior to IAV inoculation, 2 mg of antibody/mouse administered via i.p route). The data represents values from two independent experiments performed with 5 mice/group for each experiment (total 10 mice/group from two experiments); *p = 0.03. (**B**) Hematoxylin and eosin (H&E) staining of lung sections from mock infected or IAV infected mice (3×10^4^ pfu/mouse via intra-tracheal route) administered with either control IgG (IgG) or S100A9 Ab (24 h prior to IAV inoculation, 2 mg of antibody/mouse was administered via i.p route). Magnification, ×10. (**C**) Mice were administered with purified recombinant mouse S100A9 protein (15 µg/mouse) via intra-tracheal route. At 8 h post-administration, levels of mouse TNF-α in the lung was assessed by performing ELISA analysis with lung homogenate. (**D**) Lung homogenate prepared from mock infected and IAV infected (2×10^4^ pfu/mouse via intra-tracheal route) mice administered with either control IgG (IgG) or anti-S100A9 blocking (neutralizing) antibody (S100A9 Ab) (24 h prior to IAV inoculation, 2 mg of antibody/mouse administered via i.p route) were subjected to ELISA analysis to determine levels of mouse TNF-α in the lung. (**E**) For *ex-vivo* experiment, broncho-alveolar lavage fluid (BALF) was collected (at 3 d post-infection) from IAV infected mice (2×10^4^ pfu/mouse via intra-tracheal route) administered with either control IgG (IgG) or anti-S100A9 blocking (neutralizing) antibody (S100A9 Ab) (24 h prior to IAV inoculation, 2 mg of antibody/mouse administered via i.p route). The BALF cells were isolated and plated in 48-well plate. After 2 h and 4 h, the medium supernatant was analyzed for mouse TNF-α (TNF) and mouse IL-6 by ELISA. Values shown in (C), (D) and (E) represent the mean ± standard deviation from three independent experiments performed in triplicate. *p<0.05 using a Student's t test. Veh; HBSS buffer diluted in PBS (vehicle control).

Initially, we investigated the role of secreted S100A9 in regulating IAV susceptibility. For these studies, mice were i.p. injected with either control IgG or anti-S100A9 blocking antibody. One day later, mice were infected with IAV via intra-tracheal (I.T) inoculation. Survival of IAV-infected mice was monitored until 8 days postinfection. Blocking S100A9 activity significantly reduced the mortality of IAV-infected mice (Figure 9A), demonstrating that extracellular S100A9 is a key regulator of IAV susceptibility. Extracellular S100A9 also contributes to morbidity since mice treated with S100A9 blocking antibody exhibited reduced weight loss upon IAV infection (Figure S9C). Thus, extracellular S100A9 contributes to both IAV-induced mortality and morbidity.

In addition, inflammation was reduced following inhibition of extracellular S100A9 activity (Figure 9B and S10A). These results demonstrated that extracellular S100A9 contributes to the severity of IAV-associated lung inflammation and serves as a critical host factor for heightened IAV susceptibility and IAV-induced death. The clinical significance of our result is borne out by the possibility of passive immunization with anti-S100A9 antibody to reduce the severity of respiratory disease associated with IAV infection.

### Extracellular S100A9 controls the pro-inflammatory response in IAV-infected lungs

We have identified extracellular (secreted) S100A9 as a critical regulator of the pro-inflammatory response following IAV infection of macrophages. To examine the physiological role of secreted S100A9 in lung inflammation, we tested whether intratracheal (I.T.) administration of purified S100A9 protein would trigger a pro-inflammatory response in the lungs. Indeed, this led to production of TNF (Figure 9C) and IL-6 (Figure S10B) in the respiratory tract due to S100A9-mediated upregulation of TNF and IL-6 gene expression in the lung (not shown). The ability of S100A9 protein to trigger pro-inflammatory mediators in the lung is further reflected by observing airway inflammation in S100A9 protein administered (via I.T) mice (Figure S10C).

Based on this observation, we next examined the role of extracellular S100A9 in airway pro-inflammatory response following IAV infection. Mice were given i.p. injections of control IgG antibody or anti-S100A9 blocking antibody. At 1 d post-antibody treatment, mice were infected with IAV via I.T route. Levels of IL-6 and TNF in the lung were measured by ELISA. Extracellular S100A9 contributes to production of pro-inflammatory mediators during infection as evident from reduced TNF (Figure 9D) and IL-6 (Figure S11A) levels in the lung of S100A9 antibody treated mice. Reduced pro-inflammatory cytokine production was caused by loss of TNF (Figure S11B) and IL-6 (data not shown) mRNAs in the lungs of IAV-infected mice treated with S100A9 blocking antibody. Diminished pro-inflammatory response is not due to reduced IAV infection, since both control antibody and S100A9 antibody treated mice exhibited similar IAV infection status (i.e. viral burden) (Figure S12A). Interestingly, S100A9 antibody could also be utilized as therapeutics to control IAV-associated disease, since administration of S100A9 blocking antibody after IAV infection significantly reduced pro-inflammatory response and lung inflammation (Figure S12B and S12C).

In order to provide evidence for direct neutralization of S100A9 activity in the airway following i.p. administration of S100A9 antibody, we administered S100A9 antibody (via i.p.) to mice and after one day (to exactly mimic IAV infection studies) mice were inoculated with S100A9 protein via I.T route. Significant inhibition in pro-inflammatory activity was noted in the presence of S100A9 antibody (Figure S13A), which shows that i.p. administered blocking antibody can neutralize S100A9 protein in the airway.

The role of extracellular S100A9 was further validated by conducting *ex vivo* experiment with BALF-associated cells derived from IAV infected mice administered (via i.p) with either control antibody or S100A9 blocking antibody. Significant reduction in IL-6 and TNF production from BALF cells was observed in S100A9 blocking antibody treated mice (Figure 9E). This result once again validates blocking of S100A9 activity in the alveolar space localized (i.e. present in the BALF) cells. These studies illustrate the importance of secreted S100A9 in regulating pro-inflammatory cytokine gene expression and production during IAV infection of the airway.

### Extracellular S100A9 promotes apoptosis in the respiratory tract of IAV-infected mice

**Figure 10:**
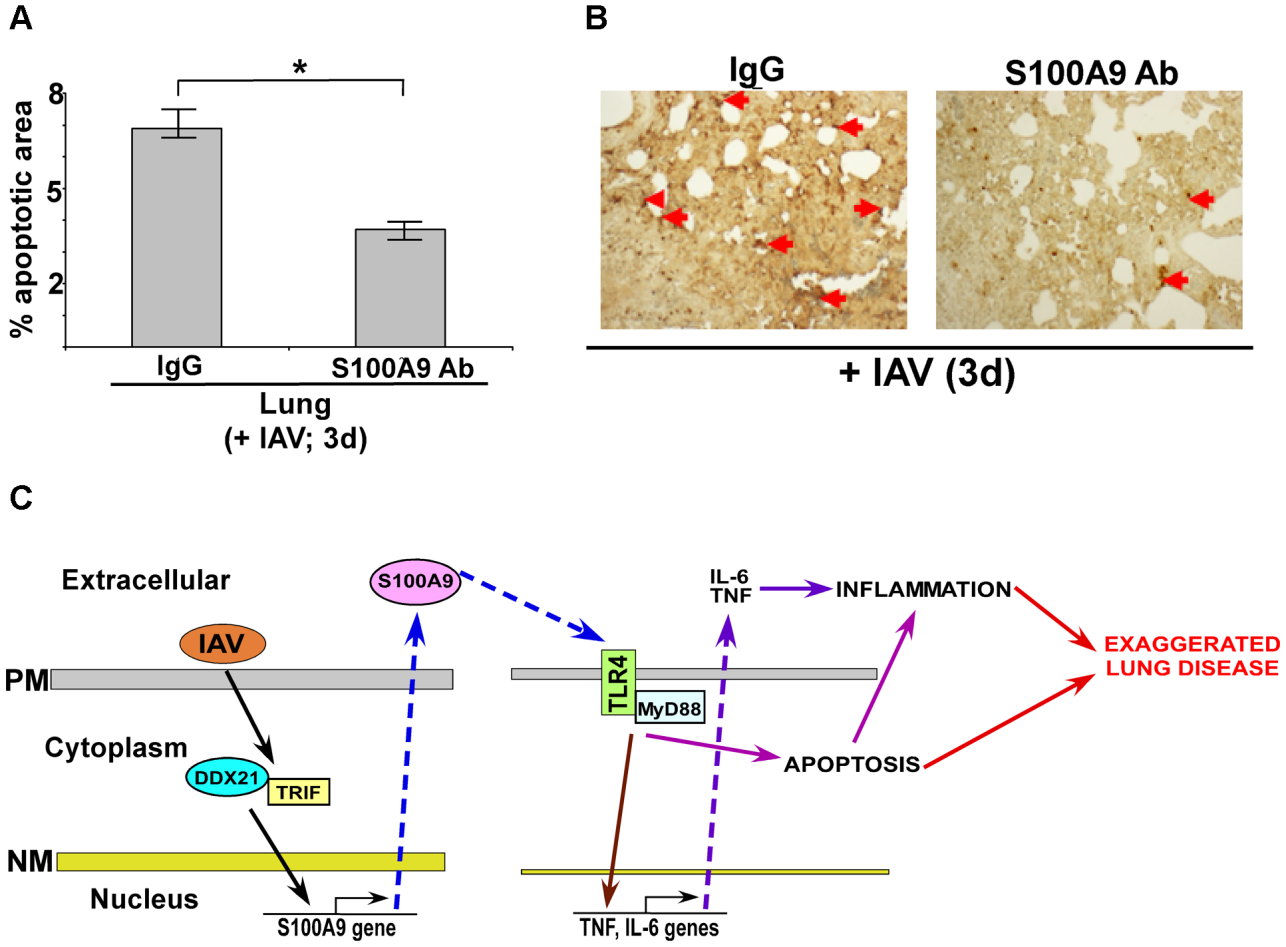
Extracellular S100A9 promotes optimal apoptosis in the lung of IAV infected mice. (**A**) Lung sections were prepared (at 3 d post-infection) from IAV infected (2×10^4^ pfu/mouse via intra-tracheal route) mice administered with either control IgG (IgG) or anti-S100A9 blocking (neutralizing) antibody (S100A9 Ab) (24 h prior to IAV inoculation, 2 mg of antibody/mouse administered via i.p route). For each experimental group lung sections were prepared from three control IgG treated mice (+IAV) and three S100A9 Ab treated mice (+IAV). The lung sections were used for TUNEL staining. Image J software was used to calculate TUNEL-positive areas (representing apoptosis) in the lung sections as detailed in the methods section. The data is presented as percent apoptotic area. The percent apoptotic area was calculated from nine areas/lung section as detailed in the methods section. The values were compiled to calculate the percent apoptotic area in IAV infected IgG treated mice vs. IAV infected S100A9 Ab treated mice, ^*^p = 0.0164 by Student's t test. (**B**) A representative TUNEL staining of lung sections from IAV infected mice administered with either IgG or S100A9 Ab. The apoptotic nuclei (representing apoptosis) are indicated with red arrows. (**C**) A schematic model depicting the role of extracellular S100A9 and DDX21/TRIF/S100A9/TLR4/MyD88 signaling network in exaggerating lung disease during IAV infection. PM, plasma membrane; NM, nuclear membrane.

Our studies with macrophages have illuminated a vital role of extracellular S100A9 in inducing apoptosis of IAV-infected cells. We have extended those observations in mice to establish the *in vivo* physiological relevance of extracellular S100A9 as a regulator of apoptosis. Further, it is known that apoptosis significantly contributes to IAV infection severity and associated lung disease [40], [63]–[68]. Therefore, reduced apoptosis in IAV-infected S100A9-blocked mice may contribute to reduced susceptibility and diminished airway disease (as shown in Figure 9A and 9B and S9C). To examine this possibility, mice treated with control IgG and S100A9 blocking antibody were inoculated with IAV via the I.T route. On the third day post-infection, we performed *in situ* TUNEL assay with lung sections to determine the apoptotic status of the IAV-infected respiratory tract. We found significantly less apoptosis in the lungs of mice given S100A9 blocking antibody than in the lungs of control mice (Figure 10A and B). These results demonstrated that secreted S100A9 is a pivotal regulator of lung apoptosis following IAV infection.

## Discussion

The role of DAMPs as a host-derived molecular pattern during virus infection is not known. In the current study we have demonstrated that extracellular S100A9 protein functions as a host-derived molecular pattern during infection. Although S100A9 is classified as a DAMP, using clinically important influenza A virus (IAV) infection model, we show release of S100A9 from “undamaged” cells during IAV infection; which triggered PRR (i.e., TLR) signaling. Surprisingly, we observed that extracellular (secreted) S100A9 regulates two key mechanisms that contribute to inflammation during IAV infection. These are pro-inflammatory cytokine production during early infection and induction of apoptosis. We also found that S100A9-mediated activation of the TLR4/MyD88 pathway resulted in increased inflammation, which culminated in exacerbated IAV pathogenesis. Thus, our study shows a role of “non-PAMP” PRR-activating DAMPs in modulating immunity and inflammation during virus infection. We also have identified DDX21-TRIF-S100A9-TLR4-MyD88 as a novel signaling “network” that regulates inflammation. It is possible that a similar pathway is used to promote disease during infection with other viruses including highly pathogenic RNA viruses like SARS, Ebola virus, Marburg virus.

Based on our results, we propose a model (Figure 10C) whereby the S100A9 gene is activated by the DDX21-TRIF pathway and the resulting S100A9 protein is secreted during IAV infection. Extracellular S100A9 activates the TLR4/MyD88 pathway via an autocrine or paracrine mechanism. As a consequence, S100A9/TLR4 activity exacerbates lung disease by promoting a pro-inflammatory response and inducing cell-death. Our studies are the first to highlight the role of S100A9 protein and DDX21-TRIF-S100A9-TLR4-MyD88 signaling network in modulating inflammation during virus infection. The clinical significance of our study is borne out by detection of S100A9 protein in mucosal secretions from IAV-infected individuals [73]. The clinical significance of utilizing neutralizing antibody is obvious from possible passive immunization with S100A9 antibody (as shown in Figure 9A and B and S9C) as a new therapeutic strategy to control lung inflammation and associated lung disease during IAV infection.

Mortality among IAV-infected individuals is associated with pneumonia, a disease characterized by massive lung inflammation leading to tissue damage and endothelial barrier disruption, resulting in fluid leakage in the airway and the development of edema. Highly pathogenic IAV strains have greater propensity to launch a hyper-inflammatory response in the respiratory tract upon infection, culminating in the development of pneumonia. Among the cellular factors that regulate IAV-induced lung disease, TLR4 is a major contributor to susceptibility and exacerbated pathophysiology associated with IAV infection [22], [23]. TLR4 is activated during IAV infection and reduced mortality and diminished lung disease (and inflammation) was observed in IAV infected TLR4 KO mice [22], [23]. The mechanism of TLR4 activation by IAV is unknown, especially since IAV doses not posses TLR4 ligand LPS. In that regard, our current study has elucidated a role of extracellular S100A9 in activation of TLR4/MyD88 pathway during IAV infection.

Previous studies reported that S100A9-S100A8 complex optimizes LPS-mediated TLR4 activation [38]. Bone marrow cells, including undifferentiated monocytes and DCs, and mice were treated/infected with LPS and LPS- expressing bacteria (E. coli 018:K1) to demonstrate augmentation of LPS activity by extracellular S100A9-S100A8 complex [38]. However, we show for the first time that S100A9 alone can directly activate TLR4 (in the absence of LPS) and contribute to the regulation of inflammation during infection with IAV, a non-LPS-expressing pathogen. Thus, extracellular S100A9 can directly modulate immune response via TLR4 activation. We also demonstrated that S100A9 alone (independent of S100A8) is a critical regulator of pro-inflammatory response *in vitro* and *in vivo*. Although few studies have been done on the mechanism of S100 gene induction during biological responses, we have shown that the S100A9 gene is induced by the DDX21-TRIF pathway. These studies also demonstrated the critical function of the DDX family PRRs in regulating expression of a host factor (S100A9) that is not a cytokine (i.e. IFNs).

We have determined that the DDX21-TRIF pathway is required for S100A9 gene expression. A recent study has shown that the DDX1-DDX21-DHX36/TRIF pathway triggers a type-I interferon response in myeloid dendritic cells (mDCs) during virus infection and treatment of cells with dsRNA (poly-IC) [43]. In these studies, direct interaction of viral dsRNA with DDX proteins was not shown. Thus, during virus infection viral dsRNA can directly or indirectly activate DDX proteins for signaling. In that context, S100A9 related S100A8 gene expression was induced by dsRNA via MAPK pathway [74].

In accord with the previous studies [43], [74], we noted that IAV replication, which will generate viral dsRNA, is required for S100A9 production, since UV inactivated IAV failed to secrete S100A9 from macrophages (Figure S13B). Surprisingly, our study demonstrated that apart from the reported DDX/TRIF-dependent IFN production in mDCs [43], the DDX/TRIF pathway is also important in inducing a pro-inflammatory response during IAV infection of macrophages, which is mediated by DDX21-TRIF dependent activation of S100A9 gene expression and resulting autocrine/paracrine action (via TLR4/MyD88 pathway) of secreted S100A9 protein. Thus, our studies have illustrated a role of two PRRs in modulating inflammation during IAV infection – a cytosolic PRR (i.e. DDX21) regulating S100A9 gene expression and membrane-localized PRR (i.e. TLR4) transducing the biological activity (i.e. pro-inflammatory response and cell death) of S100A9. This shows how concerted activity of two PRRs is used to control inflammation, since the inflammatory response has to be “regulated” at several levels due to the detrimental effect of uncontrolled inflammation on promoting cell and tissue damage.

We have also identified extracellular S100A9 as one of the host factors that regulate apoptosis during IAV infection. Apoptosis is a key contributor to pathogenesis and the pathology associated with IAV infection [40], [63]–[68], [75]. Cell death intensifies inflammation in the respiratory tract, culminating in exacerbated lung disease. Previous studies have shown the ability of S100 proteins to induce cell death by various mechanisms [51]–[55]. Similar mechanisms may contribute to S100A9-mediated cell-death following IAV infection.

Two arms of viral innate immunity consist of antiviral and inflammatory responses. Our studies have indicated that extracellular and intracellular S100A9 may function differently in terms of innate immune response during IAV infection, whereby extracellular S100A9 modulates pro-inflammatory response (independent of viral replication) and intracellular S100A9 is involved in orchestrating antiviral response to reduce viral burden. The differential activity of extracellular vs. intracellular S100A9 protein has been noted previously [69], [70]. In that regard, two different pools (i.e. extracellular and intracellular) of S100A9 exist in IAV infected macrophages (Table S1). During infection, while 10%–25% of S100A9 protein is released, the rest is localized inside the cell (Table S1). In the current study we demonstrated that extracellular S100A9 (secreted form) triggers pro-inflammatory response and apoptosis. In contrast, we speculate that intracellular S100A9 may be involved in negatively regulating antiviral response or it is required for efficient IAV infection/replication. This conclusion was based on the observation that - a) treatment of macrophages (and mice) with S100A9 blocking antibody diminished inflammatory response, while virus replication/infection was unchanged (Figure S5A, S5B and S12A), b) virus replication in S100A9 KO macrophages is reduced compared to WT cells (Figure S7A), and c) addition of S100A9 protein (to mimic extracellular S100A9 protein) to IAV infected S100A9 deficient (KO) cells led to a pro-inflammatory response even in the absence of intracellular S100A9 (i.e. in S100A9 KO BMDMs) (Fig. 4D). Thus, extracellular S100A9 regulates inflammatory response independent of virus replication, while intracellular S100A9 may negatively regulate expression/production of antiviral factor(s) or it functions as a host factor required for efficient IAV infection/replication. In the future we will further dissect the exact mechanism(s) by which intracellular S100A9 modulates IAV infection/replication.

S100A9 confers protective immunity during *Klebsiella pneumoniae* infection, since enhanced bacterial dissemination, lung damage, and susceptibility was observed in mice deficient in S100A9 expression [76]. In these studies, no distinction was made in terms of unique and differential function of extracellular vs. intracellular S100A9. However, our studies have shown that extracellular S100A9 is one of the factors that dictate detrimental host (inflammatory and apoptotic response) response during IAV infection, and this response is independent t of virus replication. In contrast, we show that intracellular S100A9 (which constitutes majority of S100A9 protein in IAV infected cells) is required for efficient IAV infection/replication. Therefore, our studies have illustrated a distinct role of extracellular vs. intracellular S100A9 during IAV infection. Thus, in accord with the previous study with *Klebsiella* we speculate that IAV infection of S100A9 KO mice will result in diminished IAV replication and as a consequence, these mice will exhibit reduced inflammation, susceptibility and pathogenesis. We will conduct these studies in the future to elucidate the exact role (and underlying mechanism) of intracellular S100A9 in controlling IAV infection/replication.

Apart from macrophages, epithelial cells (primary mouse lung epithelial cells) also produced S100A9 upon IAV infection (Figure S14). Interestingly, lower levels of S100A9 was released from primary lung epithelial cells compared to primary alveolar macrophages [please compare Figure S14 (lung epithelial cells) vs. Figure 1D (alveolar macrophages)]. In the future we plan to perform in-depth study to investigate the role of macrophages and lung epithelial cells (and their cross-talk) during S100A9 mediated inflammatory response following IAV infection.

In summary, we have identified extracellular S100A9 as a host-derived molecular pattern that regulates inflammation during virus infection. In addition, we have uncovered DDX21-TRIF-S100A9-TLR4-MyD88 as a novel signaling “network” that regulates inflammation. Future studies dealing with identification and characterization of host-derived molecular patterns (e.g. DAMPs) during virus invasion may lead to the development of measures to combat infection-associated inflammatory diseases.

## Materials and Methods

### Ethics statement

Animal studies were performed according to housing and care of laboratory animals guidelines established by National Institutes for Health. All animal experiments were reviewed and approved by the Institutional Animal Care and Use Committee (IACUC) of University of Texas Health Science Center at San Antonio. The Animal Welfare Assurance # is A3345-01.

### Virus, cell culture, mice

Influenza A [A/*PR*/8/34 (H1N1)] virus was grown in the allantoic cavities of 10-day-old embryonated eggs [21], [75]. Virus was purified by centrifuging two times on discontinuous sucrose gradients [21], [75], [77]. J774A.1 cells were maintained in DMEM supplemented with 10% fetal bovine serum (FBS), penicillin, streptomycin, and glutamine. U937 cells were maintained in RPMI 1640 medium supplemented with 10% FBS, 100 IU/mL penicillin, 100 µg/mL streptomycin, 1 mM sodium pyruvate, and 100 nM HEPES. MH-S cells were maintained in RPMI 1640 medium supplemented with 10% FBS, 100 IU/mL penicillin, and 100 µg/mL streptomycin. Bone-marrow-derived macrophages (BMDMs) were obtained from femurs and tibias of wild-type (WT) and knock-out mice and were cultured for 6–8 days as described earlier [21], [75]. Cells were plated on 12-well plates containing RPMI, 10% FBS, 100 IU/mL penicillin, 100 µg/mL streptomycin, and 20 ng/ml GM-CSF. Alveolar macrophages were obtained from the broncho-alveolar lavage fluid (BALF) of wild-type C57BL/6 mice. The IAV titer was monitored by plaque assay analysis with MDCK cells.

S100A9 KO mice were generated at University of Laval, Quebec, Canada. Other KO mice (TLR4, TLR2, TRAM, TRIF, TIRAP) were originally provided by Dr. Doug Golenbock (University of Massachusetts Medical School, Worcester, MA) under a Materials Transfer Agreement with Dr. Shizuo Akira (Osaka University, Osaka, Japan). TLR3 KO, TLR7 KO and MyD88 KO mice were obtained from Jackson Laboratory, Bar Harbor, ME.

### Antibodies and proteins

Murine S100A9 neutralizing antibody purified IgG from the serum of S100A9 immunized rabbits was generated as described previously [44]–[45]. This antibody has been successfully used to block the activity of extracellular mouse S100A9 [44]–[50]. Human S100A9 antibody was acquired from AbCam, Cambridge, MA (goat anti-human S100A9 antibody) and R&D Systems (mouse anti-human antibody). Recombinant human and mouse S100A9 proteins were generated as previously described [44]–[50]. Briefly, full length human S100A9 cDNA was cloned into pET28 expression vector (Novagen, Madison, WI). S100A9 protein expression was induced with 1 mM isopropyl β-D-thiogalactoside (IPTG) in *E. coli* HMS174 (Boehringer Mannheim, Mannheim, Germany) for 16 h at 16°C. After IPTG treatment, the bacteria were centrifuged at 5000×*g* for 10 min and the pellet was re-suspended in PBS [(containing NaCl (0.5 M) and imidazole (1 mM)] and lysed by sonication. Upon centrifuging the lysate at 55,000×*g* for 30 min at 4°C, the supernatant was collected. Recombinant His-Tag S100A9 was purified by using a nickel column. S100A9 bound to the column was incubated with 10 U of biotinylated thrombin (Novagen) (for 20 h at room temperature) to free S100A9 from its His-Tag. Recombinant S100A9 was then eluted with PBS. The digestion and elution processes were repeated one more time to cleave the remaining undigested recombinant proteins, and streptavidin-agarose (Novagen) was added to remove contaminating thrombin. Finally, the protein preparation was passed through a polymyxin B-agarose column (Pierce, Rockford, IL) to remove endotoxins.

Recombinant proteins were prepared in Hank's buffered salt solution (HBSS) buffer. The absence of endotoxin contamination in antibody and protein preparations was confirmed using the limulus amebocyte assay (Cambrex).

### Reverse transcription-PCR (RT-PCR)

Total RNA was extracted using Tri Reagent (Invitrogen). cDNA was synthesized using a High-Capacity cDNA Reverse Transcription Kit (Applied Biosystems). PCR was done using 0.25 units of *Taq* polymerase, 10 pmol of each oligonucleotide primer, 1 mM MgCl_2_, and 100 µM deoxynucleotide triphosphates in a final reaction volume of 25 µl. Following amplification, the PCR products were analyzed on 1.5% agarose gel. Equal loading in each well was confirmed by analyzing expression of the housekeeping gene glyceraldehyde-3-phosphate dehydrogenase (GAPDH). The primers used to detect the indicated genes by RT-PCR were:

*GAPDH forward,* *5′-*GTCAGTGGTGGACCTGACCT, *GAPDH reverse,* *5′-*AGGGGTCTACATGGCAACTG,

*Mouse GAPDH forward,* *5′-*GCCAAGGTCATCCATGACAACTTTGG, *Mouse GAPDH reverse,* *5′-*GCCTGCTTCACCACCTTCTTGATGTC

*Mouse S100A9 forward,* *5′-*GTCCTGGTTTGTGTCCAGGT, *Mouse S100A9 reverse,* *5′-*TCATCGACACCTTCCATCAA

*Mouse DDX21 forward,* *5′-*GATCCCCCTAAATCCAGGAA, *Mouse DDX21reverse,* *5′-*TTCGGAAGGCTCCTCTGTTA

*Mouse TNF-α forward,* *5′-*CCTGTAGCCCACGTCGTAGC, *Mouse TNF-α reverse,* *5′-*TTGACCTCAGCGCTGAGTTG

*Mouse IL-6 forward,* *5′-*TTGCCTTCTTGGGACTGATGCT, *Mouse IL-6 reverse,* *5′-*GTATCTCTCTGAAGGACTCTGG

*IAV HA forward,* *5′-* CCCAAGGAAAGTTCATGG, *IAV HA reverse,* *5′-*GAACACCCCATAGTACAAGG

### Viral infection of cells

U937 cells, alvelolar macrophages, BMDM, MH-S, and J774A.1 were infected with purified IAV [1 multiplicity of infection (MOI)−2 MOI as indicated] in serum-free, antibiotic-free OPTI-MEM medium (Gibco). Virus adsorption was done for 1.5 h at 37°C, after which cells were washed twice with PBS. Infection was continued in the presence of serum containing DMEM or RPMI medium for the specified time points.

In some experiments, cells were infected in the presence of 2 ng–10 ng/ml control IgG (purified rabbit IgG, Innovative Research, Novi, MI) or 2 ng–10 ng/ml anti-S100A9 blocking antibody. Following virus adsorption, antibodies were added to the cells and the infection was carried out in the presence of the antibodies. In addition, in some experiments infection was done in the presence of purified S100A9 protein or HBSS buffer (vehicle control). Purified S100A9 protein (5 µg/ml) was added to S100A9 KO BMDMs following virus adsorption. Purified protein was present during infection.

### siRNA

Control siRNA and mouse DDX21 siRNA were purchased from Santa Cruz Biotechnology. MH-S cells were transfected with 40 pmol of siRNAs using Lipofectamine 2000 (Invitrogen). At 48 h posttransfection, the cells were infected with IAV.

### ELISA assay

Medium supernatant and mouse lung homogenate were analyzed for TNF and IL-6 levels by using a TNF and IL-6 specific ELISA kit (eBioscience, San Diego, CA). For S100A9 ELISA, Costar High-Binding 96-well plates (Corning, NY) were coated overnight at 4°C with 800 ng/well of purified rabbit IgG against mouse S100A9 or 100 ng/well of goat polyclonal human S100A9 antibody (Abcam) diluted in 0.1 M carbonate buffer, pH 9.6. The wells were blocked with PBST+1% BSA for 1 h at room temperature. The samples were added and incubated overnight at 4°C. The plates were washed three times with PBST and incubated with either goat anti-mouse IgG (300 ng/well) (R&D) (for mouse S100A9) or mouse anti-human IgG (50 ng/well) (R&D) (for human S100A9) in PBST+0.1% BSA for 2 h at room temperature. The plates were then washed three times in PBST. To detect mouse S100A9, rabbit anti-goat HRP (Bio-Rad) was added to the plates. To detect human S100A9, goat anti-mouse HRP (Bio-Rad) was added. After 1 h incubation at room temperature, the plates were washed three times with PBST. TMB-S substrate (100 µl/well) (Sigma-Aldrich) was added to the plates according to the manufacturer's instructions. The ODs were detected at 450 nm, using a Modulas micro-plate reader.

To detect i.p.-injected S100A9 antibody in the lung homogenate, Costar High-Binding 96-well plates were coated overnight at 4°C with mouse S100A9 protein diluted in 0.1 M carbonate buffer, pH 9.6. The wells were blocked with PBST+1% BSA for 1 h at room temperature. The lung homogenate was added and incubated overnight at 4°C. The plates were washed three times with PBST and goat anti-rabbit HRP (Bio-Rad) was added. After 1 h of incubation at room temperature, the plates were washed three times with PBST. TMB-S substrate (100 µl/well) (Sigma-Aldrich) was added to the plates according to the manufacturer's instructions. ODs were detected at 450 nm by using a Modulas micro-plate reader.

### IAV infection of mice

For survival experiments, 6–8-week old pathogen-free WT C57BL/6 mice (Jackson Laboratory) were injected i.p. with 2 mg/mouse of either control IgG or anti-S100A9 antibody. One day later, mice were anesthetized and inoculated via the intratracheal or I.T route with IAV (1×10^5^ pfu/mouse) in 100 µl of PBS (Invitrogen). Control mice were sham-inoculated with 100 µl of PBS. Survival was monitored until 8 days postinfection. For pathogenesis assay, mice were inoculated with IAV (2×10^4^ pfu/mouse via the I.T route) at 1 day after antibody treatment. At 3 days after infection, lungs and BALF were collected. Lung tissue sections were used for H&E analysis and *in-situ* TUNEL analysis. Lung homogenate was used for ELISA analysis (for TNF and IL-6). RT-PCR analysis for TNF and IL-6 expression was done with RNA isolated from mouse lungs. BALF was used for Western blotting with S100A9 antibody and S100A9 ELISA analysis.

In some experiments, purified mouse S100A9 protein (15 µg/mouse) diluted in PBS or HBSS buffer diluted in PBS (vehicle control) was administered to mice via the I.T route. At 8 h posttreatment, TNF and IL-6 expression and production in the lung was monitored by RT-PCR and ELISA.

### Immunohistochemistry

Lung sections from mock- or IAV-infected mice were stained with goat anti-mouse S100A9 antibody (1∶100 dilution) (R&D) for 2 h at room temperature. After washing five times with PBS, lung sections were incubated with anti-goat Texas Red (1∶50 dilution) (Vector Labs) for 1 h at room temperature. After washing three times with PBS, sections were mounted with DAPI containing mounting solution (Invitrogen). Sections were visualized by fluorescence microscopy.

### TUNEL assay

To study apoptosis in the respiratory tract, TUNEL assays were done. Formalin-fixed lungs from IAV-infected mice were used. The TUNEL assay was done using an ApopTag Peroxidase In Situ Apoptosis Detection Kit (Milipore, MA). Digital images of TUNEL-stained lung sections were examined by light microscopy. Digital images were used to count the number of TUNEL-positive cells, using Image J software from NIH (http://rsbweb.nih.gov/ij/) as described previously by us [75]. For each analysis, an area of 5.39×10^2^ µm×4.09×10^2^ µm of TUNEL-stained lung section was scanned by Image J software. Gross apoptotic area was expressed as pixels per micron. This value was used to calculate the percentage of the apoptotic area in each analysis. Three IAV- infected mice treated with control IgG and three IAV-infected mice treated with S100A9 antibody were used. Data were collected from 9 areas per mouse from each experimental group. The values obtained from the 27 lung section areas of each experimental group were used for statistical analysis.

### H&E staining

Hematoxylin and eosin (H&E) staining was performed on paraffin-embedded mouse lung sections. Briefly, slices of lung were sequentially rehydrated in 100% and 95% ethanol followed by xylene deparaffinization. After rinsing with distilled water, sections were stained with hematoxylin for 8 min and counterstained in eosin for 1 min followed by serial dehydration with 95% and 100% ethanol. Sections were then mounted on coverslips.

### Apoptosis assay

IAV-infected and S100A9 protein-treated cells were examined for apoptosis by annexin V labeling, using an annexin V/propidium iodide (PI) apoptosis detection kit (BioVision, CA) [75], [78], [79]. For TUNEL assay cells were grown in cover slips (12 mm diameter) (Ted-Pella, CA). TUNEL assay with macrophages was performed by using DeadEnd Colorimetric TUNEL System (Promega, WI). Digital images of TUNEL-stained macrophages were examined by light microscopy. Digital images were used to count the number of TUNEL-positive cells using Image J software (please see above). At least eight different fields were counted for each cover slip and two cover slips (duplicate) were examined for each experiment. Furthermore, each experiment was repeated independently three times.

## Supporting Information

Figure S1 (**A**) U937 and J774.1 cells were infected with IAV at 1 MOI and 2 MOI, respectively. At indicated post-infection time-periods the medium supernatant was collected to assess levels of LDH by Biovision LDH kit. The value shown represents the mean ± standard deviation from three independent experiments. (**B**) RT-PCR analysis of S100A9 expression in IAV infected MH-S cells transfected with either control siRNA or DDX21 siRNA. The RT-PCR data is a representative of three independent experiments with similar results. Method: LDH assay was performed by using the LDH-Cytotoxicity Assay kit-II (BioVision, Mountain View, CA). Briefly, medium supernatant from IAV-infected macrophages were collected and centrifuged at 600×g for 10 min. After centrifugation, clear medium supernatant was incubated with LDH Reaction Mix for 15 min at room temperature to assess levels of LDH by measuring the absorbance at 450 nm, using a Modulas micro-plate reader. Percentages of cytotoxicity was calculated according to manufacturer's instructions and values of background control (i.e. medium only) were subtracted from all other values.
(PDF)

Figure S2 (**A**) RT-PCR analysis of IAV hemagglutinin (HA) expression in IAV infected MH-S cells transfected with either control siRNA or DDX21 siRNA. (**B**) RT-PCR analysis of IAV HA expression in infected wild type (WT) and TRIF knockout (KO) bone marrow derived macrophages (BMDM). The RT-PCR gel shown in (A) and (B) is a representative of three independent experiments with similar results. BMDMs isolated from WT and MyD88 KO (**C**) or MAVS KO (**D**) mice were infected with IAV. At 24 h post-infection time-period the medium supernatant was collected to assess levels of S100A9 protein by ELISA. The value shown in (C) and (D) represents the mean ± standard deviation from three independent experiments performed in triplicate. p value shown in the figure was derived by using Student's t test.
(PDF)

Figure S3 J774A.1 macrophages were incubated with purified recombinant mouse S100A9 protein (5 ug/mL) for 6 h and 12 h. RT-PCR analysis was performed to detect expression of mouse IL-6 (**A**) and mouse TNF-α(TNF) (**B**). Each RT-PCR data is a representative of three independent experiments with similar results. Vehicle; cells incubated with HBSS buffer (vehicle control).
(PDF)

Figure S4 Purified recombinant S100A9 protein was incubated with polymyxin B (10 µg/mL) for 2 h. S100A9 protein was also heat inactivated at 80°C for 30 min. Polymyxin treated and heat inactivated S100A9 protein (5 ug/mL) was then added to primary bone marrow derived macrophages (BMDM) to assess IL-6 (**A**) and TNF (**B**) production by ELISA. (**C**) TNF production from mouse J774A.1 macrophages infected with IAV (2 MOI) in the presence of either control IgG (IgG) or anti-S100A9 blocking (neutralizing) antibody (S100A9 Ab) was analyzed by ELISA. (**D**) IL-6 production from wild-type BMDM infected with IAV in the presence of either control IgG or S100A9 Ab was analyzed by ELISA. (**E**) RT-PCR analysis of IL-6 expression in IAV infected BMDM treated with either control IgG or S100A9 Ab. The RT-PCR data is a representative of three independent experiments with similar results. The values shown in (A), (B), (C) and (D) represent the mean ± standard deviation from three independent experiments performed in triplicate. *p<0.05 using a Student's t test.
(PDF)

Figure S5 (**A**) RT-PCR analysis of IAV hemagglutinin (HA) expression in mouse J774A.1 macrophages infected with IAV (2 MOI) in the presence of either control IgG (IgG) or anti-S100A9 blocking (neutralizing) antibody (S100A9 Ab). (**B**) RT-PCR analysis of IAV HA expression in wild-type (WT) bone marrow derived macrophages (BMDM) infected with IAV in the presence of either control IgG or S100A9 Ab. (**C**) RT-PCR analysis of TNF-α expression in IAV infected WT and S100A9 knockout (KO) BMDMs. Each RT-PCR data is a representative of three independent experiments with similar results.
(PDF)

Figure S6 Role of S100A9 during pro-inflammatory response mediated by poly-IC, TNF and imiquimod. (**A**) Primary bone marrow derived macrophages (BMDM) were treated with 5 µg/ml poly-IC (Invivogen, San Diego, CA). At indicated post-treatment time-periods the medium supernatant was collected to assess levels of S100A9 protein by ELISA. The values shown represent the mean ± standard deviation from three independent experiments performed in triplicate. *p<0.05 using a Student's t test. BMDM isolated from wild-type (WT) and S100A9 knockout (KO) mice were treated with poly-IC (10 µg/ml). At indicated post-treatment time-periods the medium supernatant was collected to assess levels of mouse IL-6 (**B**) and TNF-α (**C**) by ELISA. S100A9 KO BMDMs were treated with poly-IC (for 24 h) in the presence of purified recombinant mouse S100A9 protein (5 µg/ml). Medium supernatant was collected from treated cells to assess levels of mouse IL-6 (**D**) and TNF-α (**E**) by ELISA. Vehicle control cells (veh) were incubated with HBSS buffer. The values shown in (B), (C), (D) and (E) represent the mean ± standard deviation from three independent experiments performed in triplicate. *p<0.05 using a Student's t test. WT and S100A9 KO BMDMs were treated with either imiquimod (1 µg/ml for 12 h) (Invivogen, San Diego, CA) (**F and G**) or mouse TNF-α (10 ng/ml for 12 h) (R&D Systems, Minneapolis, MN) (**H**). After treatment, the medium supernatant was collected to assess levels of mouse IL-6 (**F and H**) and TNF-α (**G**) by ELISA. The values shown in (F), (G) and (H) represent the mean ± standard deviation from three independent experiments performed in triplicate. No significant difference in WT vs. KO cells was observed. UT; untreated (i.e. treated with vehicle control). For poly-IC and imiquimod treatment, sterile endogenous-free water (provided by Invivogen, San Diego, CA) served as the vehicle control; while PBS served as the vehicle control for TNF treatment.
(PDF)

Figure S7 (**A**) RT-PCR analysis of IAV HA expression in infected WT and S100A9 KO BMDM. RT-PCR data is a representative of three independent experiments with similar results. (**B**) Mouse J774A.1 macrophages were incubated with purified recombinant mouse S100A9 protein (5 µg/ml) for 48 h and 72 h. The apoptotic state of these cells was examined by TUNEL analysis. TUNEL positive cells were analyzed by image J software. Percent TUNEL positive cells denotes ratio of number of TUNEL positive cells/total number of cells. (**C**) Mouse alveolar macrophage MH-S cell-line was incubated with purified S100A9 protein (5 µg/ml). The apoptotic status was determined as described in (B). (**D**) Mouse J774A.1 macrophages were infected with IAV (2 MOI) in the presence of either control IgG (IgG) or anti-S100A9 blocking (neutralizing) antibody (S100A9 Ab). At 24 h post-infection, the apoptotic state of these cells was determined as described in (B). The values represents mean ± standard deviation from three independent experiments, *p<0.05 by Student's t test. Veh; cells incubated with HBSS buffer (vehicle control).
(PDF)

Figure S8 (**A**) RT-PCR analysis of TNF expression in S100A9 protein treated wild-type (WT) and TLR4 knockout (KO) bone marrow derived macrophages (BMDM). (**B**) RT-PCR analysis of TNF expression in S100A9 protein treated WT and MyD88 KO BMDMs. (**C**) RT-PCR analysis of IL-6 expression in IAV infected WT and TLR4 KO BMDMs. (**D**) RT-PCR analysis of IAV hemagglutinin (HA) expression in IAV infected WT and TLR4 KO BMDMs. Each RT-PCR data is a representative of three independent experiments with similar results.
(PDF)

Figure S9 (**A**) S100A9 production in the airway is not inhibited by S100A9 blocking antibody-Broncho-alveolar lavage fluid (BALF) isolated from IAV infected (2×10^4^ pfu/mouse via intra-tracheal route) mice administered with either control IgG (IgG) or anti-S100A9 blocking (neutralizing) antibody (S100A9 Ab) (24 h prior to IAV inoculation, 2 mg of antibody/mouse administered via i.p route) were subjected to ELISA analysis to determine levels of S100A9 protein in BALF. The values represent the mean ± standard deviation from three independent experiments performed in triplicate. (**B**) Presence of S100A9 antibody in the lung of mice administered with S100A9 blocking antibody via intra-peritoneal route- Lung homogenate prepared from IAV infected (2×10^4^ pfu/mouse via intra-tracheal route) mice administered with either IgG or S100A9 Ab (24 h prior to IAV inoculation, 2 mg of antibody/mouse administered via i.p route) were subjected to ELISA analysis to determine levels of S100A9 antibody. The values represent the mean ± standard deviation from three independent experiments performed in triplicate. (**C**) Body weight of IAV infected (100 pfu/mouse) mice (n = 7 mice/group) administered with either IgG or S100A9 Ab (24 h prior to IAV inoculation, 2 mg of antibody/mouse administered via i.p route). The result represents mean ± s.e.m. p<0.05 (IgG treated mice vs. S100A9 Ab treated mice) based on Student's t test. The body weight of mock infected mice treated with either IgG or S100A9 Ab were monitored every 2 d (till 18 d post-antibody treatment). None of the mice lost weight (data not shown). Method: To detect inter-peritoneal administered S100A9 antibody in the lung homogenate, Costar High Binding 96-well plates (Corning, NY) were coated overnight at 4°C with mouse S100A9 protein diluted in 0.1 M carbonate buffer, pH 9.6. The wells were blocked with PBST+1% BSA for 1 h at room temperature. The lung homogenate was added and incubated overnight at 4°C. The plates were then washed three times with PBST, followed by addition of goat anti-rabbit HRP (Bio-Rad). Following 1 h incubation at room temperature, the plates were washed three times with PBST. TMB-S substrate (100 µl/well) (Sigma-Aldrich) was added to the plates according to the manufacturer's instructions. The ODs were detected at 450 nm by using Modulas micro-plate reader.
(PDF)

Figure S10 (**A**) A magnified version of the H&E stained lung section from Figure 9B. Lung sections were obtained from IAV infected mice administered with either control IgG (IgG) or anti-S100A9 blocking (neutralizing) antibody (S100A9 Ab). (**B**) Mice were administered with purified recombinant mouse S100A9 protein (15 µg/mouse) via intra-tracheal route. At 8 h post-administration, levels of mouse IL-6 in the lung was assessed by performing ELISA analysis with lung homogenate. The values represents mean ± standard deviation from three independent experiments, *p<0.05 by Student's t test. Veh; cells incubated with HBSS buffer (vehicle control). (**C**) H&E staining of lung sections from mice administered (via intra-tracheal route) with either vehicle or purified recombinant mouse S100A9 protein (15 µg/mouse).
(PDF)

Figure S11 (**A**) Lung homogenate prepared from mock infected and IAV infected (2×10^4^ pfu/mouse via intra-tracheal route) mice administered with either control IgG (IgG) or anti-S100A9 blocking (neutralizing) antibody (S100A9 Ab) (24 h prior to IAV inoculation, 2 mg of antibody/mouse administered via i.p route) were subjected to ELISA analysis to determine levels of mouse IL-6 in the lung. The values represents mean ± standard deviation from three independent experiments, *p<0.05 by Student's t test. (**B**) RT-PCR analysis of TNF expression in the lung of IAV infected mice treated with either control IgG or S100A9 Ab. RT-PCR data represents two mock mice/group and three infected mice/group and the data is a representative of three independent experiments with similar results.
(PDF)

Figure S12 (**A**) Blocking extracellular S100A9 does not affect IAV burden in the lung - IAV infectious titer in the lung homogenate of infected mice (3×10^4^ pfu/mouse via intra-tracheal route) administered with either control IgG (IgG) or anti-S100A9 blocking (neutralizing) antibody (S100A9 Ab) (24 h prior to IAV inoculation, 2 mg of antibody/mouse was administered via i.p route) was assessed at 3 d post-infection by plaque assay analysis. The viral titer represents the mean ± standard deviation from three independent experiments performed in triplicate. p value (p = 0.1) shown in the figure was derived by using Student's t test. (**B and C**) Therapeutic activity of S100A9 blocking antibody – (**B**) Lung homogenate prepared from mock infected and IAV infected (2×10^4^ pfu/mouse via intra-tracheal or I.T route) mice administered with either IgG or S100A9 Ab (3 h after IAV inoculation, 420 µg of antibody/mouse administered via I.T route) were subjected to ELISA analysis to determine levels of mouse TNF-α in the lung. The values represents mean ± standard deviation from three independent experiments, *p<0.05 by Student's t test. (**C**) H&E staining of lung sections from mock infected and IAV infected mice administered with either IgG or S100A9 Ab (3 h after IAV inoculation, 420 µg of antibody/mouse administered via I.T route).
(PDF)

Figure S13 (**A**) S100A9 antibody administered via intra-peritoneal route blocks pro-inflammatory activity of S100A9 in the airway - Mice were administered with either control IgG (IgG) or anti-S100A9 blocking (neutralizing) antibody (S100A9 Ab) (2 mg of antibody/mouse was administered via i.p route). After 24 h, mice were administered with purified recombinant mouse S100A9 protein (15 µg/mouse) via intra-tracheal route. At 8 h post-S100A9 protein administration, levels of mouse TNF-α in the lung was assessed by performing ELISA analysis with lung homogenate. The values represents mean ± standard deviation from three independent experiments, *p<0.05 by Student's t test. (**B**) S100A9 production from MH-S cells infected with either UV-IAV (UV-irradiated IAV virus) or control-IAV (non UV irradiated IAV virus) for 12 h and 24 h. S100A9 was measured by ELISA analysis of medium supernatant. The values represent the mean ± standard deviation from two independent experiments performed in triplicate,*p<0.05 using a Student's t test.
(PDF)

Figure S14 Release of S100A9 protein from IAV infected primary mouse lung epithelial cells. Mouse epithelial cells isolated from the lung of C57BL/6 mice were plated and infected with IAV. At 8 h and 16 post-infection, medium supernatant was collected to assess levels of S100A9 protein by ELISA. The values shown represent the mean ± standard deviation from three independent experiments performed in triplicate. *p<0.05 using a Student's t test. Method: Lung (alveolar) epithelial cells were isolated from mouse lung as described previously (Borchers, MT et al. *J Clin Invest*. 119:636–49, 2009; Rice, WR et al. *Am. J. Physiol. Lung Cell Mol. Physiol.* 283:L256–L264, 2002). Sternohyoid muscles of the neck were removed from anesthetized mice to expose the trachea. The lungs were perfused (by using barrel-tip needle) with 6 ml of sterile PBS. The enzyme dispase (3 ml) (StemCell Technologies) was instilled through a cannula in the trachea, followed by injection of 1 ml of 1% low melt agarose. Following 2 min incubation of the mice on ice (to solidify the agarose), the lungs were removed and further incubated with 1 ml dispase for 45 min (at room temperature). The lungs were then transferred to a culture dish containing 8 ml DMEM medium (with 20 mM HEPES and 100 units/ml of penicillin and streptomycin). The lungs were then gently teased away from cell suspension and agitated for 10 min on ice. The cell suspension was filtered successively through 100 µm, 40 µm, and 20 µm nylon gauze. The suspension was then centrifuged for 5 min at 130 g. The cell pellet was re-suspended in complete DMEM medium (DMEM supplemented with 10% fetal bovine serum (FBS), penicillin, streptomycin, and glutamine). The re-suspended cells were added to dishes pre-coated with 45 µg/ml of anti-mouse CD45 (eBiosciene, CA) and CD16/32 (eBioscience, CA) antibodies. After 2 h incubation (at 37°C in the CO2 incubator), the non-adherent cells were collected and centrifuged for 5 min at 130 g. The cell pellet was re-suspended in complete DMEM and transferred to a six-well plate. Light microscopy showed that the adhered cells possess epithelial morphology. These cells which constitute primary lung (alveolar) epithelial cells were infected with IAV (2 MOI) and medium supernatant was collected to assess S100A9 levels by ELISA.
(PDF)

Table S1 Extracellular vs. intracellular S100A9 protein levels in influenza A virus (IAV) infected bone marrow derived macrophages. S100A9 protein levels were measured by ELISA. The values represent the mean ± standard deviation from three independent experiments performed in triplicate. The levels of extracellular (medium supernatant) vs. intracellular (cell lysate) S100A9 was compared. Significantly less (based on the p value of <0.05 using a Student's t test) S100A9 was present in the medium supernatant compared to the cell lysate. Percent extracellular vs. intracellular was calculated based on the ratio of S100A9 protein levels in medium supernatant or cell lysate/total S100A9 protein (i.e. the sum of S100A9 present in medium supernatant+S100A9 present in the cell lysate). We failed to detect any extracellular (in the medium supernatant) S100A9 in mock infected macrophages (data not shown).
(PDF)
