# Supplementary figures and images for: DAMP Molecule S100A9 Acts as a Molecular Pattern to Enhance Inflammation during Influenza A Virus Infection: Role of DDX21-TRIF-TLR4-MyD88 Pathway
Source: PLoS Pathog. 2014 Jan 2;10(1):e1003848. doi: 10.1371/journal.ppat.1003848 (PMC3879357; doi:10.1371/journal.ppat.1003848)

# Supp Fig. 1

A.

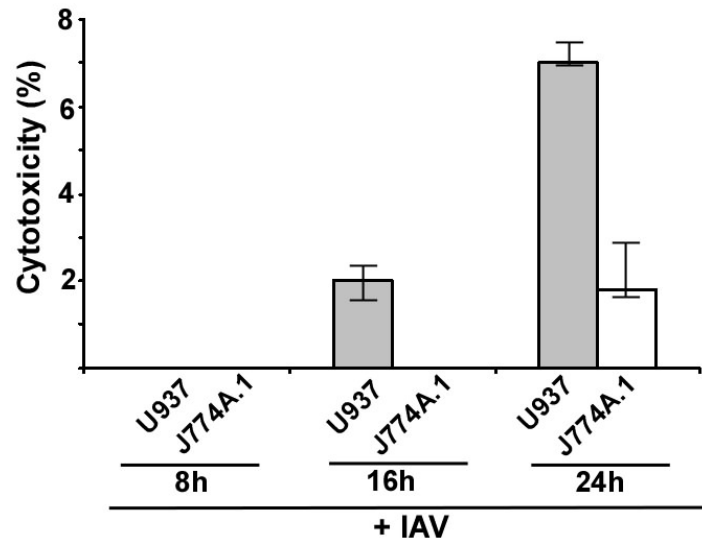

B.

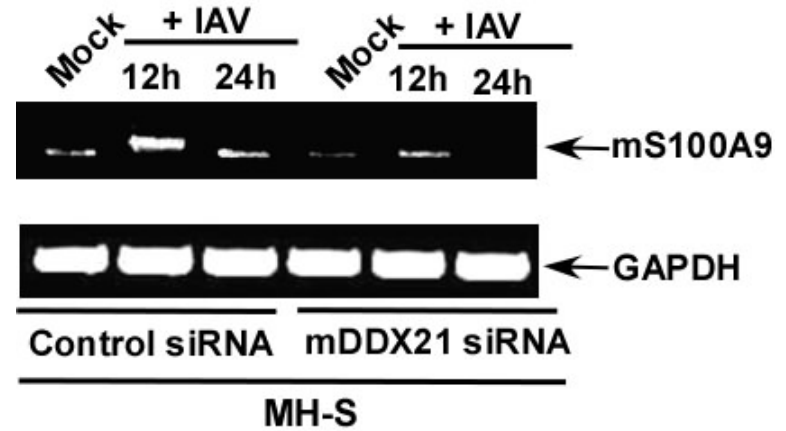

Supplement: Figure S1 — (A) U937 and J774.1 cells were infected with IAV at 1 MOI and 2 MOI, respectively. At indicated post-infection time-periods the medium supernatant was collected to assess levels of LDH by Biovision LDH kit. The value shown represents the mean ± standard deviation from three independent experiments. (B) RT-PCR analysis of S100A9 expression in IAV infected MH-S cells transfected with either control siRNA or DDX21 siRNA. The RT-PCR data is a representative of three independent experiments with similar results. Method: LDH assay was performed by using the LDH-Cytotoxicity Assay kit-II (BioVision, Mountain View, CA). Briefly, medium supernatant from IAV-infected macrophages were collected and centrifuged at 600×g for 10 min. After centrifugation, clear medium supernatant was incubated with LDH Reaction Mix for 15 min at room temperature to assess levels of LDH by measuring the absorbance at 450 nm, using a Modulas micro-plate reader. Percentages of cytotoxicity was calculated according to manufacturer's instructions and values of background control (i.e. medium only) were subtracted from all other values. (PDF) [file ppat.1003848.s001.pdf]

## Supp Fig. 2

A.

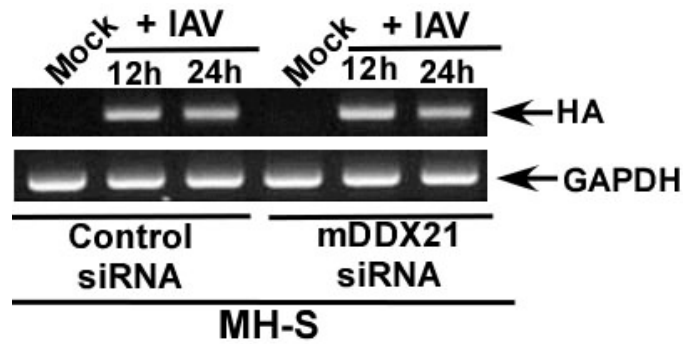

B.

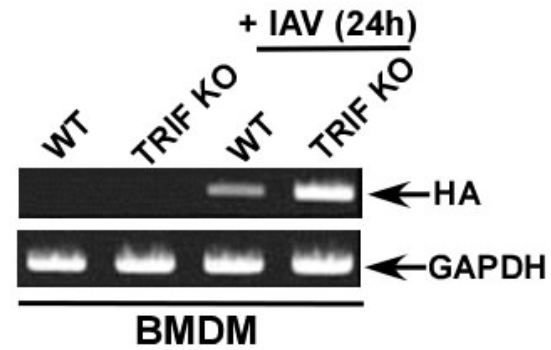

C.

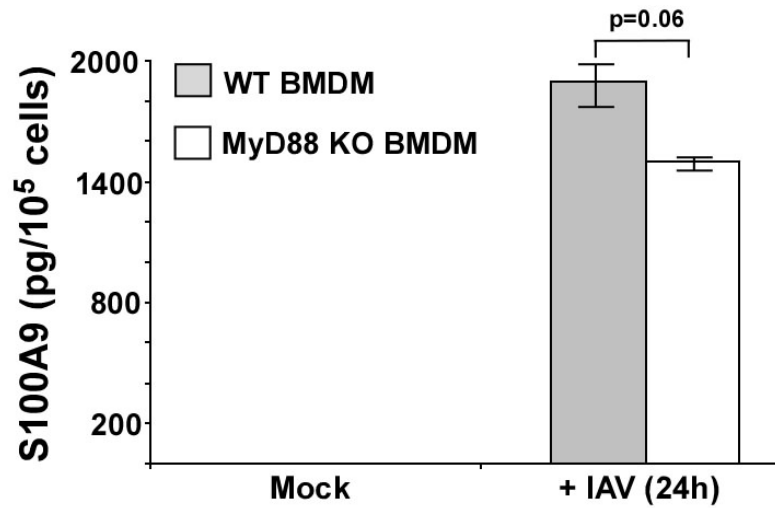

D.

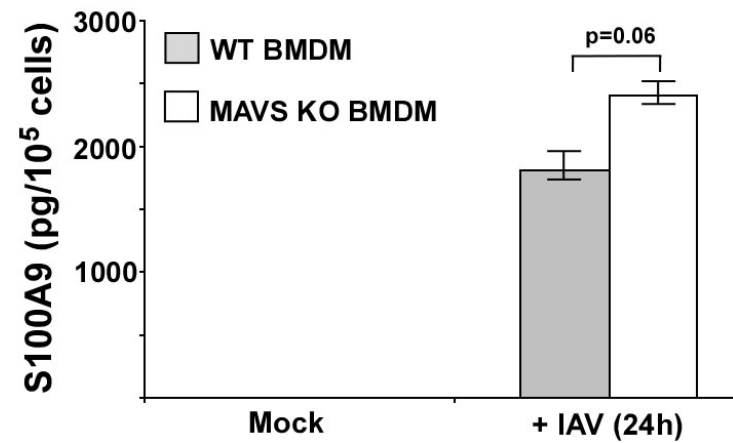

Supplement: Figure S2 — (A) RT-PCR analysis of IAV hemagglutinin (HA) expression in IAV infected MH-S cells transfected with either control siRNA or DDX21 siRNA. (B) RT-PCR analysis of IAV HA expression in infected wild type (WT) and TRIF knockout (KO) bone marrow derived macrophages (BMDM). The RT-PCR gel shown in (A) and (B) is a representative of three independent experiments with similar results. BMDMs isolated from WT and MyD88 KO (C) or MAVS KO (D) mice were infected with IAV. At 24 h post-infection time-period the medium supernatant was collected to assess levels of S100A9 protein by ELISA. The value shown in (C) and (D) represents the mean ± standard deviation from three independent experiments performed in triplicate. p value shown in the figure was derived by using Student's t test. (PDF) [file ppat.1003848.s002.pdf]

## Supp Fig. 3

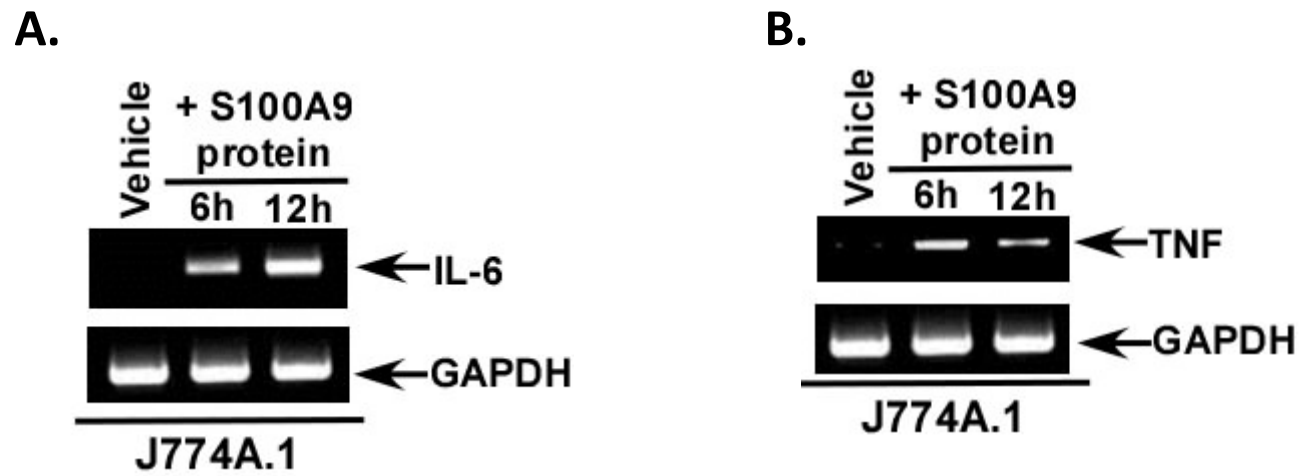

Supplement: Figure S3 — J774A.1 macrophages were incubated with purified recombinant mouse S100A9 protein (5 ug/mL) for 6 h and 12 h. RT-PCR analysis was performed to detect expression of mouse IL-6 (A) and mouse TNF-α(TNF) (B). Each RT-PCR data is a representative of three independent experiments with similar results. Vehicle; cells incubated with HBSS buffer (vehicle control). (PDF) [file ppat.1003848.s003.pdf]

# Supp Fig. 4

**A.**

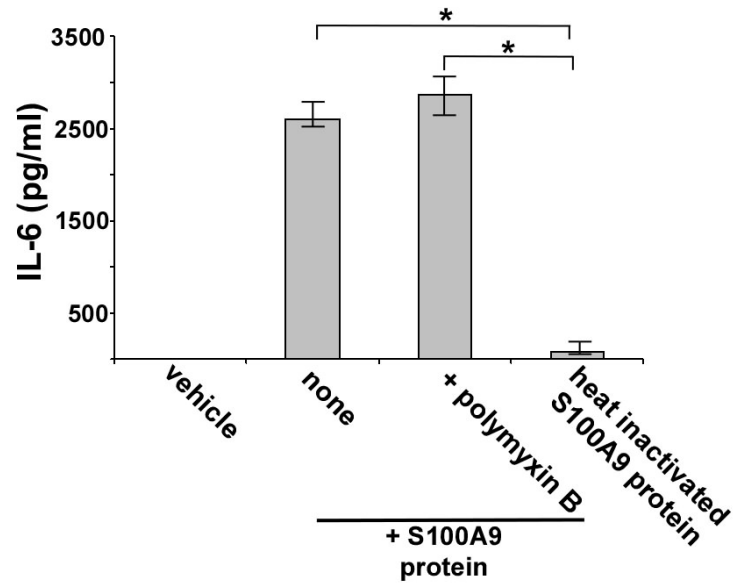

**B.**

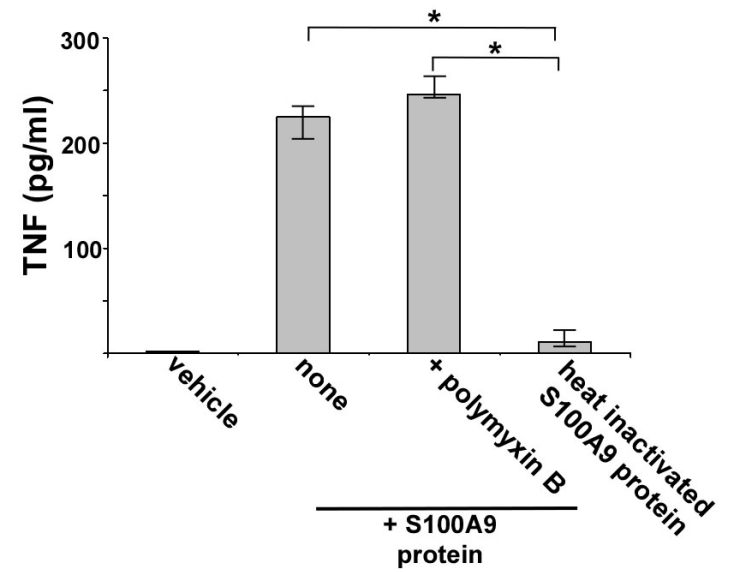

**C.**

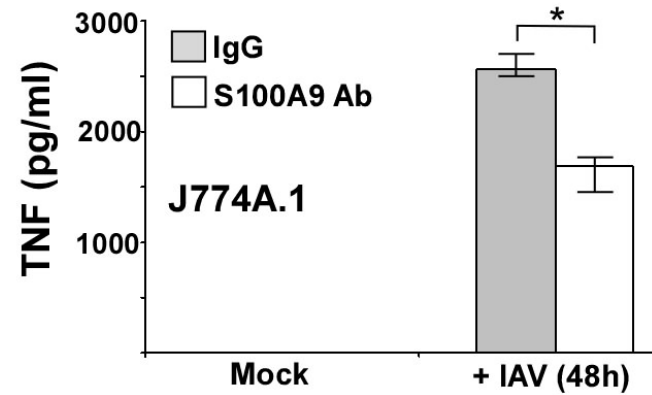

**D.**

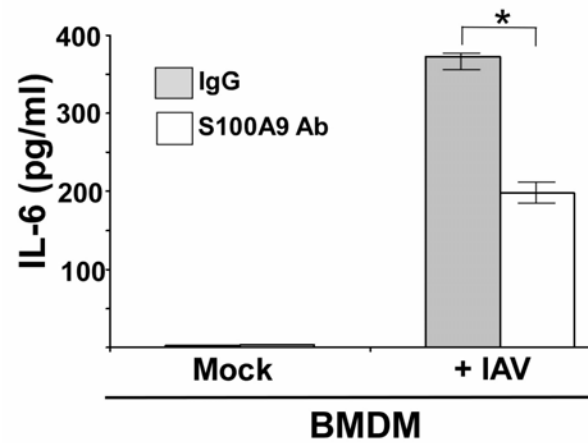

**E.**

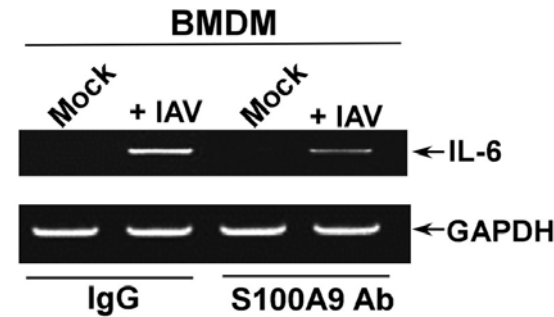

Supplement: Figure S4 — Purified recombinant S100A9 protein was incubated with polymyxin B (10 µg/mL) for 2 h. S100A9 protein was also heat inactivated at 80°C for 30 min. Polymyxin treated and heat inactivated S100A9 protein (5 ug/mL) was then added to primary bone marrow derived macrophages (BMDM) to assess IL-6 (A) and TNF (B) production by ELISA. (C) TNF production from mouse J774A.1 macrophages infected with IAV (2 MOI) in the presence of either control IgG (IgG) or anti-S100A9 blocking (neutralizing) antibody (S100A9 Ab) was analyzed by ELISA. (D) IL-6 production from wild-type BMDM infected with IAV in the presence of either control IgG or S100A9 Ab was analyzed by ELISA. (E) RT-PCR analysis of IL-6 expression in IAV infected BMDM treated with either control IgG or S100A9 Ab. The RT-PCR data is a representative of three independent experiments with similar results. The values shown in (A), (B), (C) and (D) represent the mean ± standard deviation from three independent experiments performed in triplicate. *p<0.05 using a Student's t test. (PDF) [file ppat.1003848.s004.pdf]

# Supp Fig. 5

A.

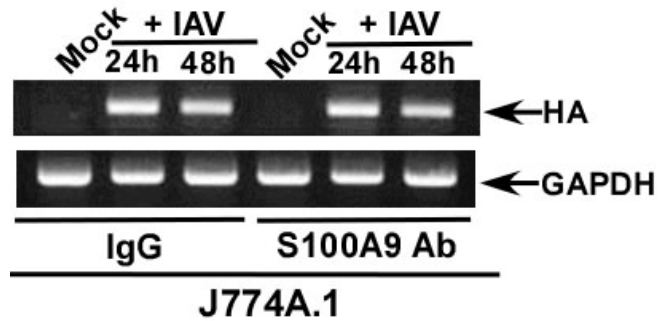

B.

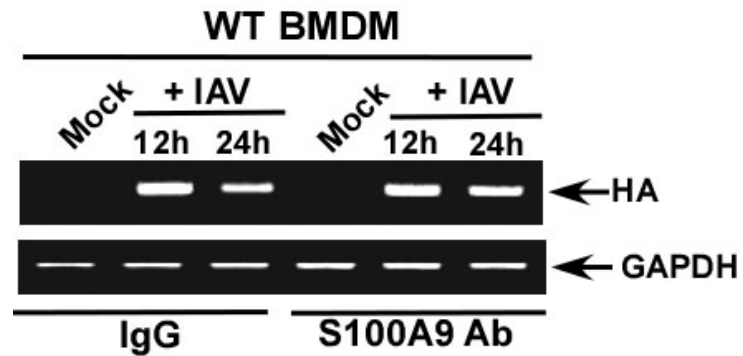

C.

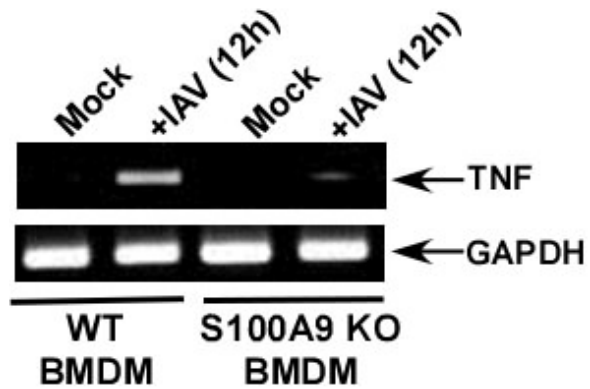

Supplement: Figure S5 — (A) RT-PCR analysis of IAV hemagglutinin (HA) expression in mouse J774A.1 macrophages infected with IAV (2 MOI) in the presence of either control IgG (IgG) or anti-S100A9 blocking (neutralizing) antibody (S100A9 Ab). (B) RT-PCR analysis of IAV HA expression in wild-type (WT) bone marrow derived macrophages (BMDM) infected with IAV in the presence of either control IgG or S100A9 Ab. (C) RT-PCR analysis of TNF-α expression in IAV infected WT and S100A9 knockout (KO) BMDMs. Each RT-PCR data is a representative of three independent experiments with similar results. (PDF) [file ppat.1003848.s005.pdf]

# Supp Fig. 6

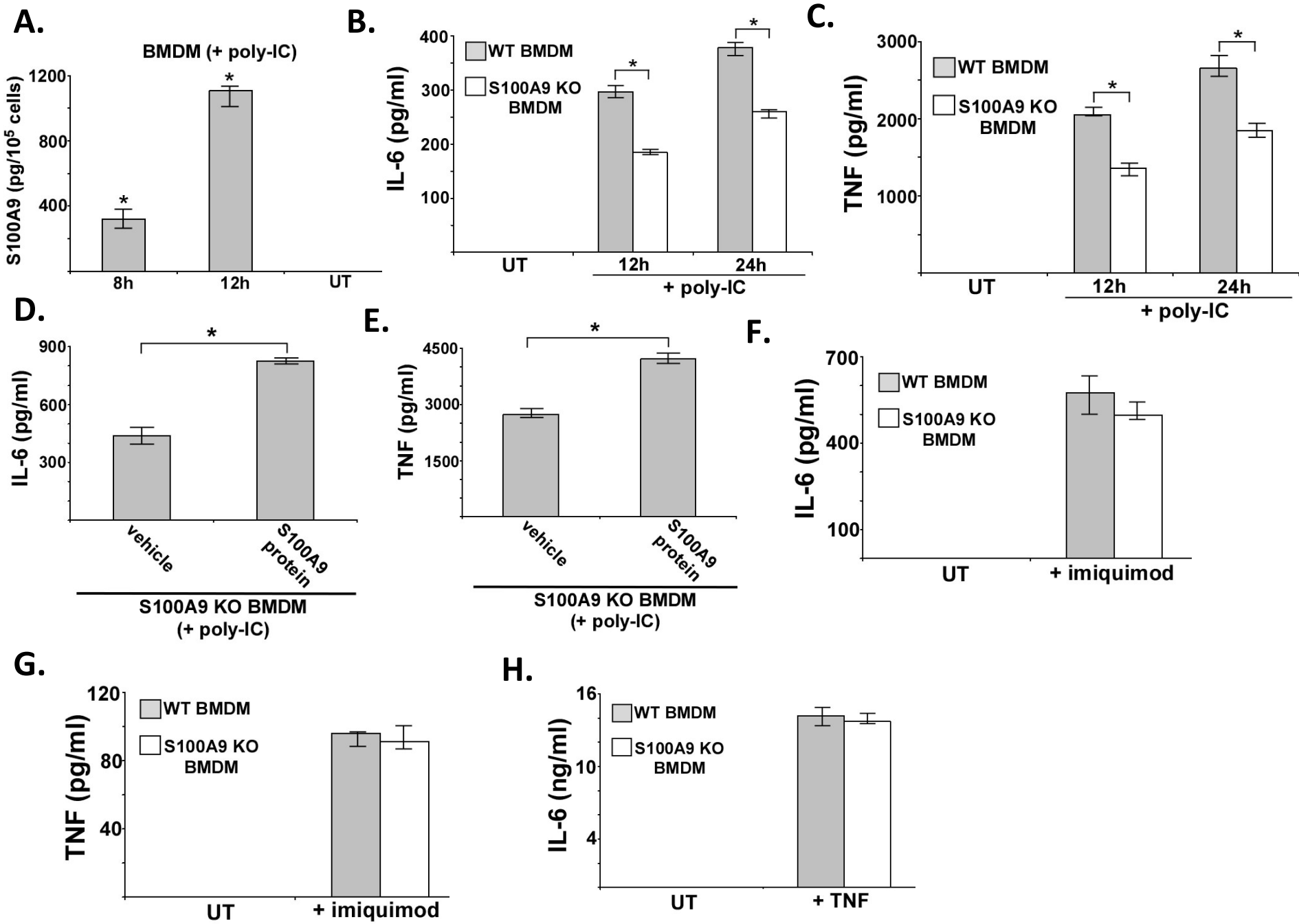

Supplement: Figure S6 — Role of S100A9 during pro-inflammatory response mediated by poly-IC, TNF and imiquimod. (A) Primary bone marrow derived macrophages (BMDM) were treated with 5 µg/ml poly-IC (Invivogen, San Diego, CA). At indicated post-treatment time-periods the medium supernatant was collected to assess levels of S100A9 protein by ELISA. The values shown represent the mean ± standard deviation from three independent experiments performed in triplicate. *p<0.05 using a Student's t test. BMDM isolated from wild-type (WT) and S100A9 knockout (KO) mice were treated with poly-IC (10 µg/ml). At indicated post-treatment time-periods the medium supernatant was collected to assess levels of mouse IL-6 (B) and TNF-α (C) by ELISA. S100A9 KO BMDMs were treated with poly-IC (for 24 h) in the presence of purified recombinant mouse S100A9 protein (5 µg/ml). Medium supernatant was collected from treated cells to assess levels of mouse IL-6 (D) and TNF-α (E) by ELISA. Vehicle control cells (veh) were incubated with HBSS buffer. The values shown in (B), (C), (D) and (E) represent the mean ± standard deviation from three independent experiments performed in triplicate. *p<0.05 using a Student's t test. WT and S100A9 KO BMDMs were treated with either imiquimod (1 µg/ml for 12 h) (Invivogen, San Diego, CA) (F and G) or mouse TNF-α (10 ng/ml for 12 h) (R&D Systems, Minneapolis, MN) (H). After treatment, the medium supernatant was collected to assess levels of mouse IL-6 (F and H) and TNF-α (G) by ELISA. The values shown in (F), (G) and (H) represent the mean ± standard deviation from three independent experiments performed in triplicate. No significant difference in WT vs. KO cells was observed. UT; untreated (i.e. treated with vehicle control). For poly-IC and imiquimod treatment, sterile endogenous-free water (provided by Invivogen, San Diego, CA) served as the vehicle control; while PBS served as the vehicle control for TNF treatment. (PDF) [file ppat.1003848.s006.pdf]

# Supp Fig. 7

A.

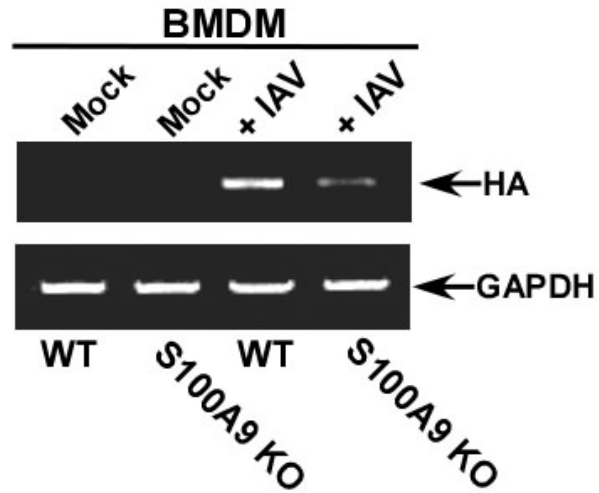

B.

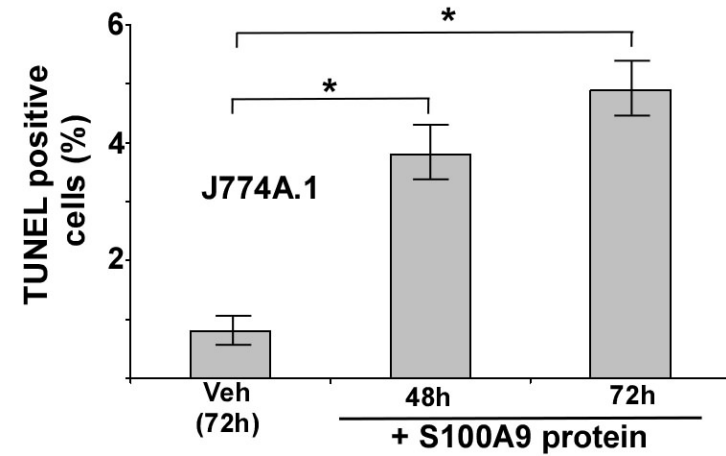

C.

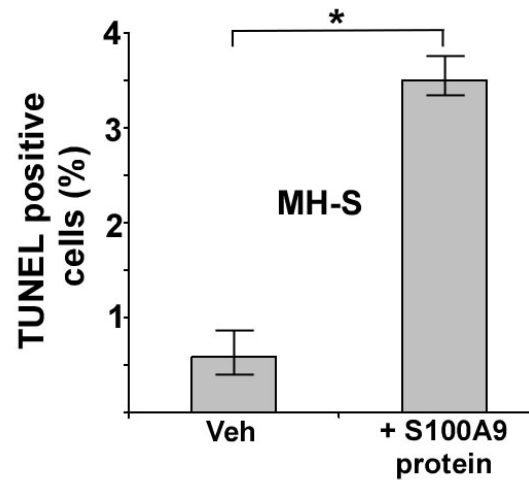

D.

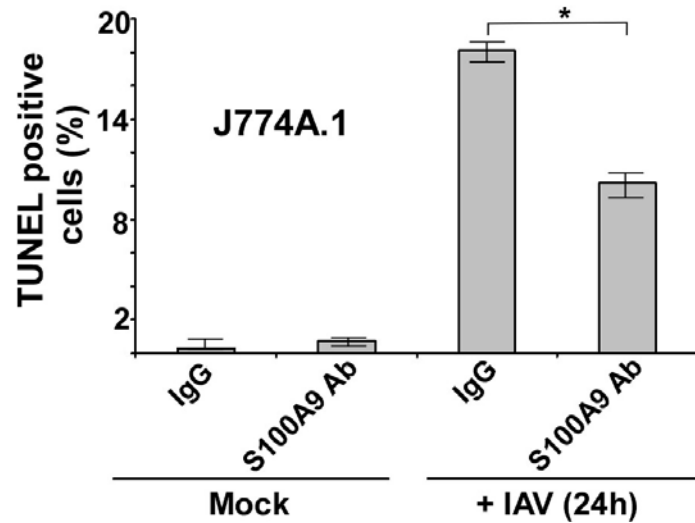

Supplement: Figure S7 — (A) RT-PCR analysis of IAV HA expression in infected WT and S100A9 KO BMDM. RT-PCR data is a representative of three independent experiments with similar results. (B) Mouse J774A.1 macrophages were incubated with purified recombinant mouse S100A9 protein (5 µg/ml) for 48 h and 72 h. The apoptotic state of these cells was examined by TUNEL analysis. TUNEL positive cells were analyzed by image J software. Percent TUNEL positive cells denotes ratio of number of TUNEL positive cells/total number of cells. (C) Mouse alveolar macrophage MH-S cell-line was incubated with purified S100A9 protein (5 µg/ml). The apoptotic status was determined as described in (B). (D) Mouse J774A.1 macrophages were infected with IAV (2 MOI) in the presence of either control IgG (IgG) or anti-S100A9 blocking (neutralizing) antibody (S100A9 Ab). At 24 h post-infection, the apoptotic state of these cells was determined as described in (B). The values represents mean ± standard deviation from three independent experiments, *p<0.05 by Student's t test. Veh; cells incubated with HBSS buffer (vehicle control). (PDF) [file ppat.1003848.s007.pdf]

# Supp Fig. 8

A.

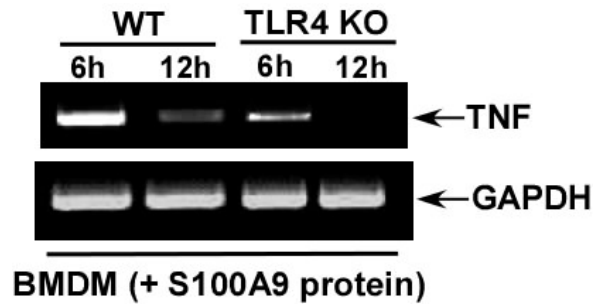

B.

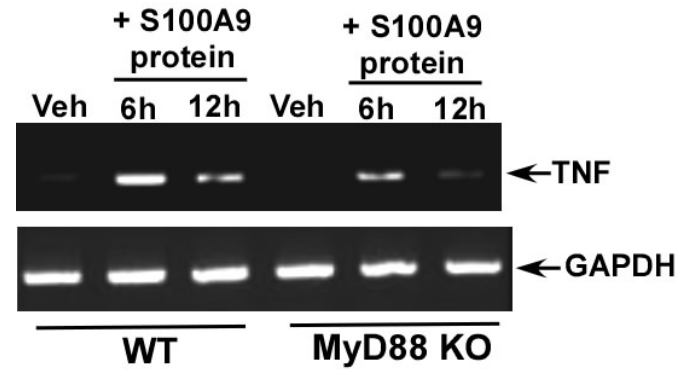

C.

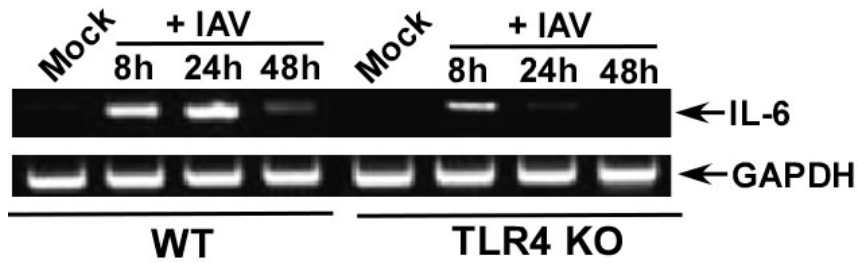

D.

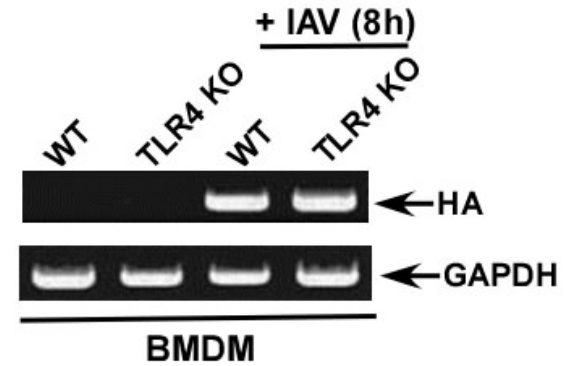

Supplement: Figure S8 — (A) RT-PCR analysis of TNF expression in S100A9 protein treated wild-type (WT) and TLR4 knockout (KO) bone marrow derived macrophages (BMDM). (B) RT-PCR analysis of TNF expression in S100A9 protein treated WT and MyD88 KO BMDMs. (C) RT-PCR analysis of IL-6 expression in IAV infected WT and TLR4 KO BMDMs. (D) RT-PCR analysis of IAV hemagglutinin (HA) expression in IAV infected WT and TLR4 KO BMDMs. Each RT-PCR data is a representative of three independent experiments with similar results. (PDF) [file ppat.1003848.s008.pdf]

## Supp Fig. 9

A.

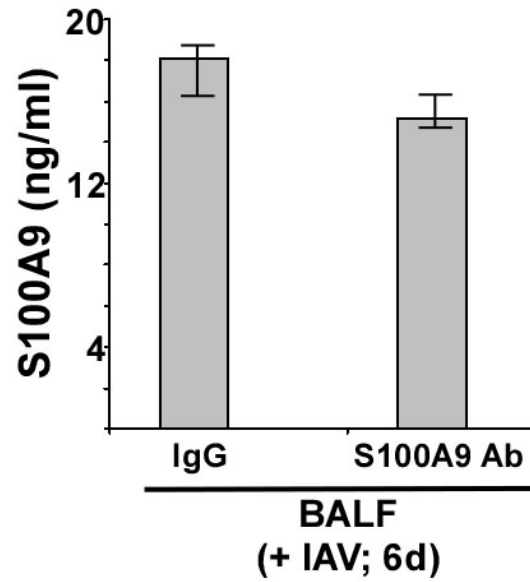

B.

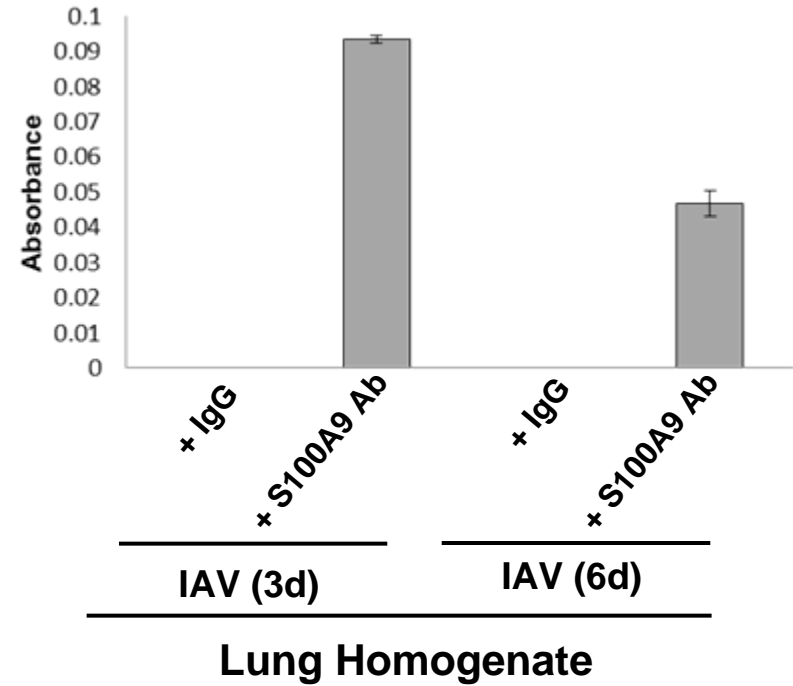

C.

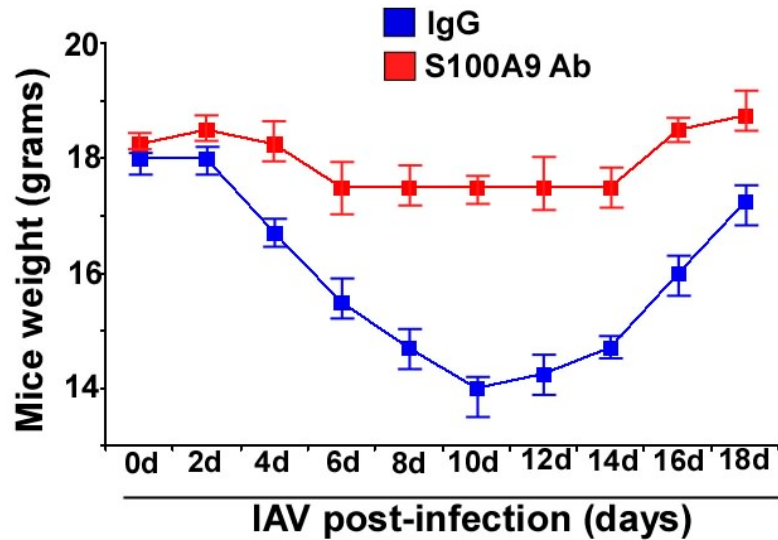

Supplement: Figure S9 — (A) S100A9 production in the airway is not inhibited by S100A9 blocking antibody-Broncho-alveolar lavage fluid (BALF) isolated from IAV infected (2×10^4^ pfu/mouse via intra-tracheal route) mice administered with either control IgG (IgG) or anti-S100A9 blocking (neutralizing) antibody (S100A9 Ab) (24 h prior to IAV inoculation, 2 mg of antibody/mouse administered via i.p route) were subjected to ELISA analysis to determine levels of S100A9 protein in BALF. The values represent the mean ± standard deviation from three independent experiments performed in triplicate. (B) Presence of S100A9 antibody in the lung of mice administered with S100A9 blocking antibody via intra-peritoneal route- Lung homogenate prepared from IAV infected (2×10^4^ pfu/mouse via intra-tracheal route) mice administered with either IgG or S100A9 Ab (24 h prior to IAV inoculation, 2 mg of antibody/mouse administered via i.p route) were subjected to ELISA analysis to determine levels of S100A9 antibody. The values represent the mean ± standard deviation from three independent experiments performed in triplicate. (C) Body weight of IAV infected (100 pfu/mouse) mice (n = 7 mice/group) administered with either IgG or S100A9 Ab (24 h prior to IAV inoculation, 2 mg of antibody/mouse administered via i.p route). The result represents mean ± s.e.m. p<0.05 (IgG treated mice vs. S100A9 Ab treated mice) based on Student's t test. The body weight of mock infected mice treated with either IgG or S100A9 Ab were monitored every 2 d (till 18 d post-antibody treatment). None of the mice lost weight (data not shown). Method: To detect inter-peritoneal administered S100A9 antibody in the lung homogenate, Costar High Binding 96-well plates (Corning, NY) were coated overnight at 4°C with mouse S100A9 protein diluted in 0.1 M carbonate buffer, pH 9.6. The wells were blocked with PBST+1% BSA for 1 h at room temperature. The lung homogenate was added and incubated overnight at 4°C. The plates were then washed three times wit [file ppat.1003848.s009.pdf]

## Supp Fig. 10

A.

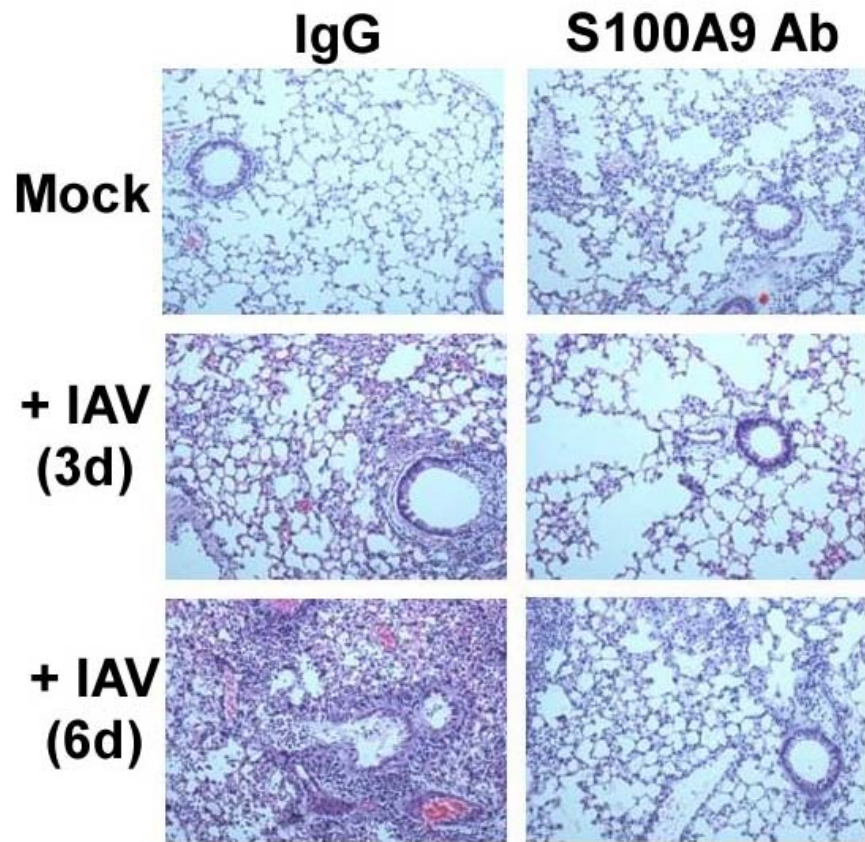

B.

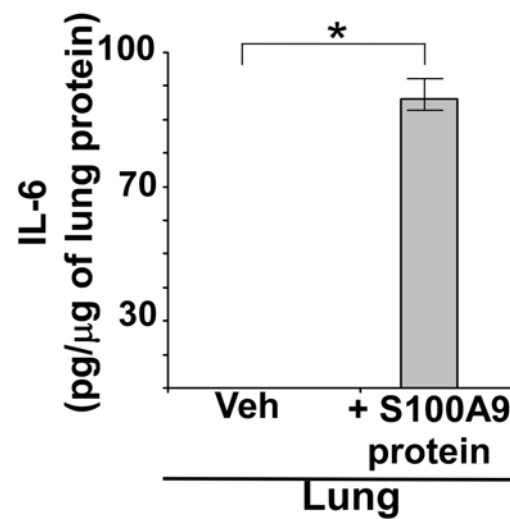

C.

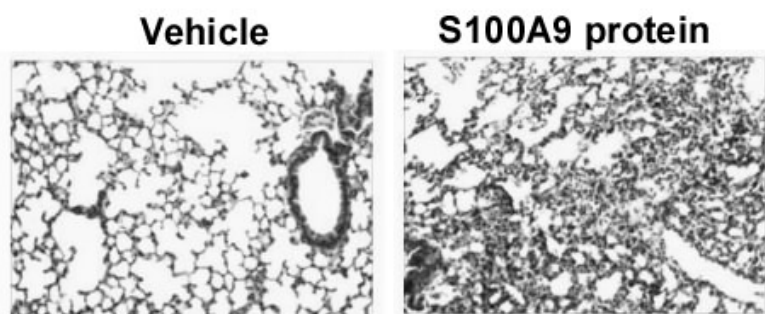

Supplement: Figure S10 — (A) A magnified version of the H&E stained lung section from Figure 9B. Lung sections were obtained from IAV infected mice administered with either control IgG (IgG) or anti-S100A9 blocking (neutralizing) antibody (S100A9 Ab). (B) Mice were administered with purified recombinant mouse S100A9 protein (15 µg/mouse) via intra-tracheal route. At 8 h post-administration, levels of mouse IL-6 in the lung was assessed by performing ELISA analysis with lung homogenate. The values represents mean ± standard deviation from three independent experiments, *p<0.05 by Student's t test. Veh; cells incubated with HBSS buffer (vehicle control). (C) H&E staining of lung sections from mice administered (via intra-tracheal route) with either vehicle or purified recombinant mouse S100A9 protein (15 µg/mouse). (PDF) [file ppat.1003848.s010.pdf]

# Supp Fig. 11

**A.**

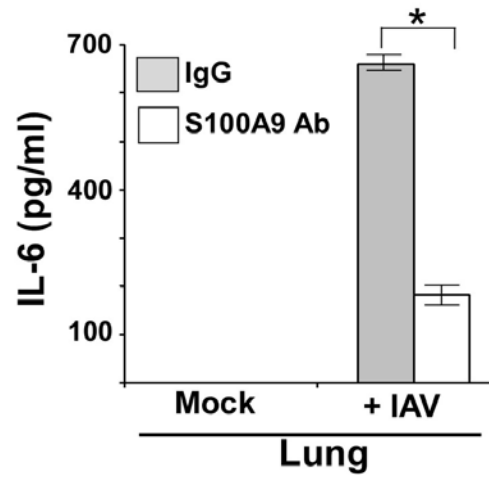

**B.**

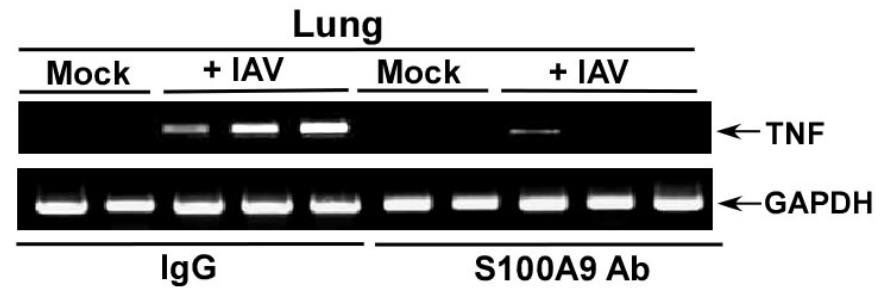

Supplement: Figure S11 — (A) Lung homogenate prepared from mock infected and IAV infected (2×10^4^ pfu/mouse via intra-tracheal route) mice administered with either control IgG (IgG) or anti-S100A9 blocking (neutralizing) antibody (S100A9 Ab) (24 h prior to IAV inoculation, 2 mg of antibody/mouse administered via i.p route) were subjected to ELISA analysis to determine levels of mouse IL-6 in the lung. The values represents mean ± standard deviation from three independent experiments, *p<0.05 by Student's t test. (B) RT-PCR analysis of TNF expression in the lung of IAV infected mice treated with either control IgG or S100A9 Ab. RT-PCR data represents two mock mice/group and three infected mice/group and the data is a representative of three independent experiments with similar results. (PDF) [file ppat.1003848.s011.pdf]

## Supp Fig. 12

**A.**

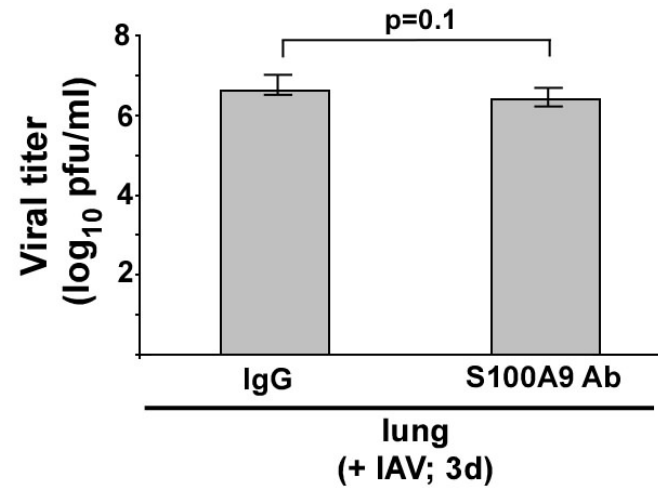

**B.**

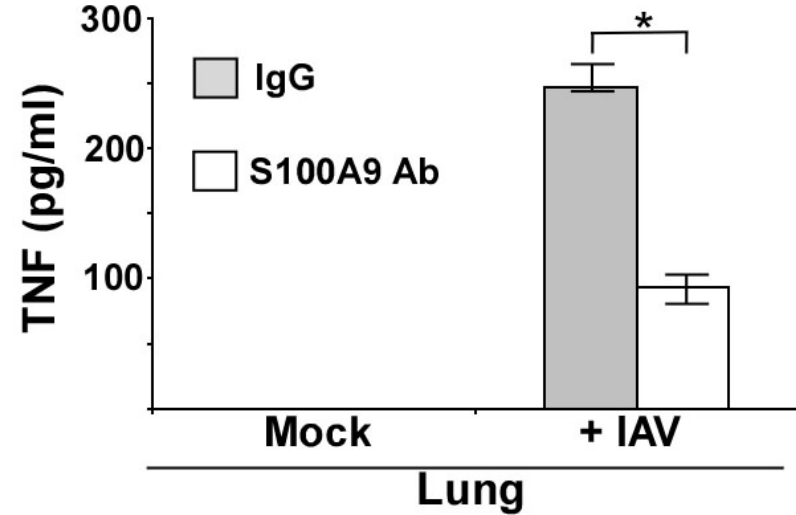

**C.**

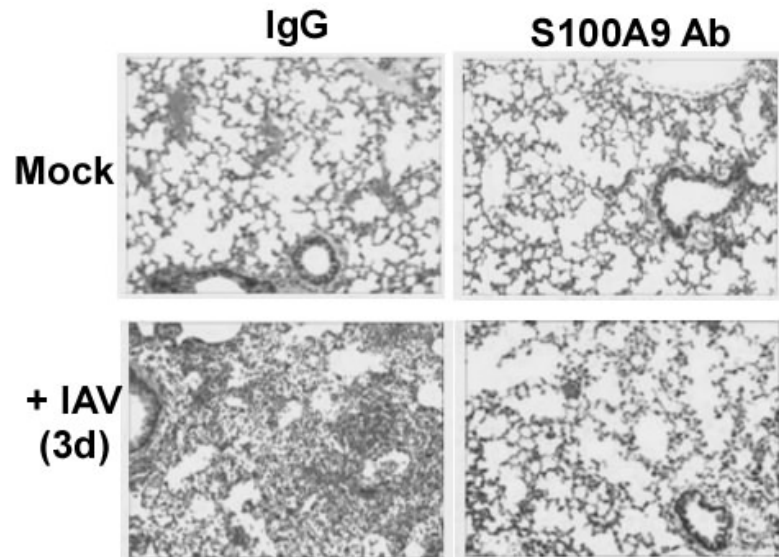

Supplement: Figure S12 — (A) Blocking extracellular S100A9 does not affect IAV burden in the lung - IAV infectious titer in the lung homogenate of infected mice (3×10^4^ pfu/mouse via intra-tracheal route) administered with either control IgG (IgG) or anti-S100A9 blocking (neutralizing) antibody (S100A9 Ab) (24 h prior to IAV inoculation, 2 mg of antibody/mouse was administered via i.p route) was assessed at 3 d post-infection by plaque assay analysis. The viral titer represents the mean ± standard deviation from three independent experiments performed in triplicate. p value (p = 0.1) shown in the figure was derived by using Student's t test. (B and C) Therapeutic activity of S100A9 blocking antibody – (B) Lung homogenate prepared from mock infected and IAV infected (2×10^4^ pfu/mouse via intra-tracheal or I.T route) mice administered with either IgG or S100A9 Ab (3 h after IAV inoculation, 420 µg of antibody/mouse administered via I.T route) were subjected to ELISA analysis to determine levels of mouse TNF-α in the lung. The values represents mean ± standard deviation from three independent experiments, *p<0.05 by Student's t test. (C) H&E staining of lung sections from mock infected and IAV infected mice administered with either IgG or S100A9 Ab (3 h after IAV inoculation, 420 µg of antibody/mouse administered via I.T route). (PDF) [file ppat.1003848.s012.pdf]

# Supp Fig. 13

A.

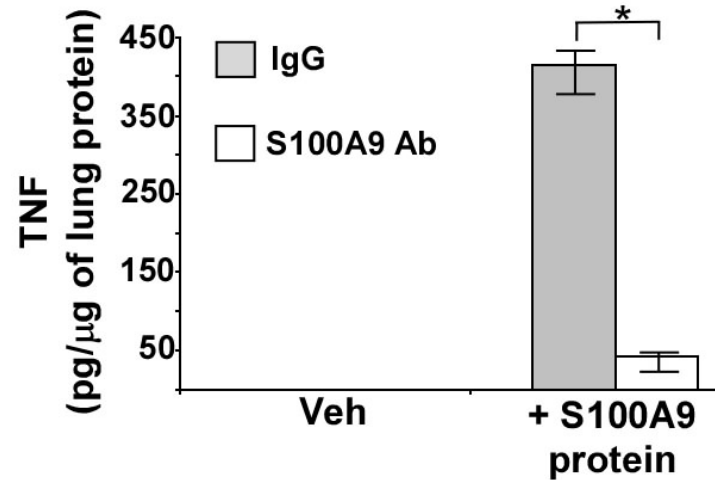

B.

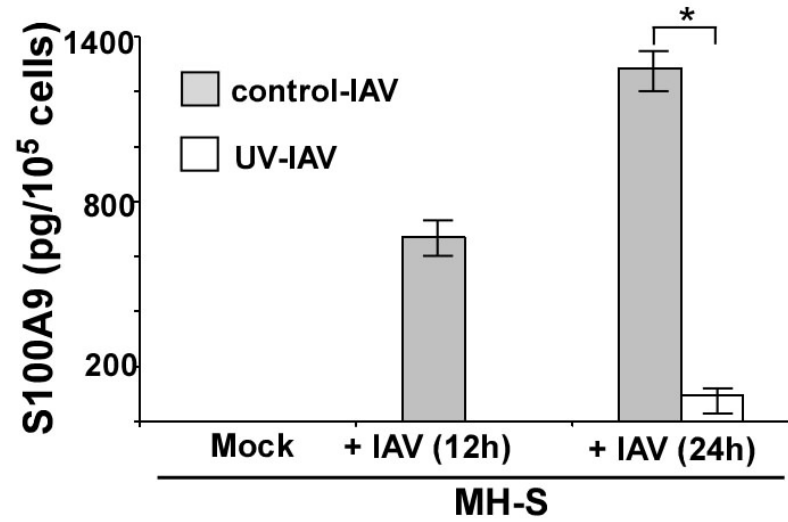

Supplement: Figure S13 — (A) S100A9 antibody administered via intra-peritoneal route blocks pro-inflammatory activity of S100A9 in the airway - Mice were administered with either control IgG (IgG) or anti-S100A9 blocking (neutralizing) antibody (S100A9 Ab) (2 mg of antibody/mouse was administered via i.p route). After 24 h, mice were administered with purified recombinant mouse S100A9 protein (15 µg/mouse) via intra-tracheal route. At 8 h post-S100A9 protein administration, levels of mouse TNF-α in the lung was assessed by performing ELISA analysis with lung homogenate. The values represents mean ± standard deviation from three independent experiments, *p<0.05 by Student's t test. (B) S100A9 production from MH-S cells infected with either UV-IAV (UV-irradiated IAV virus) or control-IAV (non UV irradiated IAV virus) for 12 h and 24 h. S100A9 was measured by ELISA analysis of medium supernatant. The values represent the mean ± standard deviation from two independent experiments performed in triplicate,*p<0.05 using a Student's t test. (PDF) [file ppat.1003848.s013.pdf]

# Supp Fig. 14

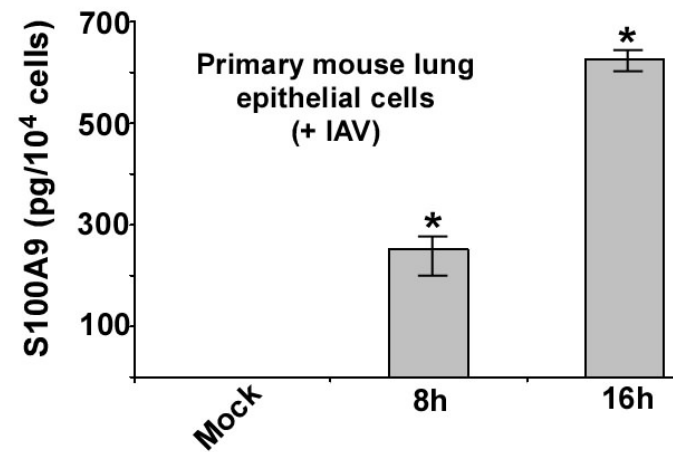

Supplement: Figure S14 — Release of S100A9 protein from IAV infected primary mouse lung epithelial cells. Mouse epithelial cells isolated from the lung of C57BL/6 mice were plated and infected with IAV. At 8 h and 16 post-infection, medium supernatant was collected to assess levels of S100A9 protein by ELISA. The values shown represent the mean ± standard deviation from three independent experiments performed in triplicate. *p<0.05 using a Student's t test. Method: Lung (alveolar) epithelial cells were isolated from mouse lung as described previously (Borchers, MT et al. J Clin Invest. 119:636–49, 2009; Rice, WR et al. Am. J. Physiol. Lung Cell Mol. Physiol. 283:L256–L264, 2002). Sternohyoid muscles of the neck were removed from anesthetized mice to expose the trachea. The lungs were perfused (by using barrel-tip needle) with 6 ml of sterile PBS. The enzyme dispase (3 ml) (StemCell Technologies) was instilled through a cannula in the trachea, followed by injection of 1 ml of 1% low melt agarose. Following 2 min incubation of the mice on ice (to solidify the agarose), the lungs were removed and further incubated with 1 ml dispase for 45 min (at room temperature). The lungs were then transferred to a culture dish containing 8 ml DMEM medium (with 20 mM HEPES and 100 units/ml of penicillin and streptomycin). The lungs were then gently teased away from cell suspension and agitated for 10 min on ice. The cell suspension was filtered successively through 100 µm, 40 µm, and 20 µm nylon gauze. The suspension was then centrifuged for 5 min at 130 g. The cell pellet was re-suspended in complete DMEM medium (DMEM supplemented with 10% fetal bovine serum (FBS), penicillin, streptomycin, and glutamine). The re-suspended cells were added to dishes pre-coated with 45 µg/ml of anti-mouse CD45 (eBiosciene, CA) and CD16/32 (eBioscience, CA) antibodies. After 2 h incubation (at 37°C in the CO2 incubator), the non-adherent cells were collected and centrifuged for 5 min at 130 g. The cell pellet was re-suspende [file ppat.1003848.s014.pdf]
