# Supplementary material for: DAMP Molecule S100A9 Acts as a Molecular Pattern to Enhance Inflammation during Influenza A Virus Infection: Role of DDX21-TRIF-TLR4-MyD88 Pathway
Source: PLoS Pathog. 2014 Jan 2;10(1):e1003848. doi: 10.1371/journal.ppat.1003848 (PMC3879357; doi:10.1371/journal.ppat.1003848)
Supplement: Table S1 — Extracellular vs. intracellular S100A9 protein levels in influenza A virus (IAV) infected bone marrow derived macrophages. S100A9 protein levels were measured by ELISA. The values represent the mean ± standard deviation from three independent experiments performed in triplicate. The levels of extracellular (medium supernatant) vs. intracellular (cell lysate) S100A9 was compared. Significantly less (based on the p value of <0.05 using a Student's t test) S100A9 was present in the medium supernatant compared to the cell lysate. Percent extracellular vs. intracellular was calculated based on the ratio of S100A9 protein levels in medium supernatant or cell lysate/total S100A9 protein (i.e. the sum of S100A9 present in medium supernatant+S100A9 present in the cell lysate). We failed to detect any extracellular (in the medium supernatant) S100A9 in mock infected macrophages (data not shown). (PDF) [file ppat.1003848.s015.pdf]

Supplemental Table S1 Extracellular vs. intracellular S100A9 protein levels in influenza A virus (IAV) infected bone marrow derived macrophages

IAV post-infection (h)	Medium supernatant (pg/ml)	Cell lysate (pg/ml)	p value	Extracellular (%)	Intracellular (%)
**8h**	1780±82	16053±1772	$p<0.05$	9.98%	90.02%
**12h**	2839±149	15054±1895	$p<0.05$	15.8%	84.2%
**24h**	4262±221	13426±1398	$p<0.05$	24%	76%

S100A9 protein levels were measured by ELISA. The values represent the mean ± standard deviation from three independent experiments performed in triplicate. The levels of extracellular (medium supernatant) vs. intracellular (cell lysate) S100A9 was compared. Significantly less (based on the p value of <0.05 using a Student's t test) S100A9 was present in the medium supernatant compared to the cell lysate. Percent extracellular vs. intracellular was calculated based on the ratio of S100A9 protein levels in medium supernatant or cell lysate/total S100A9 protein (i.e. the sum of S100A9 present in medium supernatant + S100A9 present in the cell lysate).

We failed to detect any extracellular (in the medium supernatant) S100A9 in mock infected macrophages (data not shown).
